# Association of reallocating time between physical activity and sedentary behavior on the risk of depression: a systematic review and meta-analysis

**DOI:** 10.3389/fpsyg.2025.1505061

**Published:** 2025-04-30

**Authors:** Yue Wang, Jun Sun, Yuheng Zhang, Jiali Wang, Songtao Lu

**Affiliations:** ^1^Faculty of Artificial Intelligence in Education, Central China Normal University, Wuhan, China; ^2^School of Physical Education, Central China Normal University, Wuhan, China; ^3^School of Sports, Wuhan University of Science and Technology, Wuhan, China

**Keywords:** physical activity, sedentary behavior, reallocating, depression, meta-analysis

## Abstract

**Background and aims:**

Sedentary behavior (SB) is a prevalent lifestyle factor and a risk factor for various health conditions, including depression (encompassing both clinically diagnosed depressive disorders and depressive symptoms). This study aimed to summarize the estimated impact of reallocating time spent in SB to light-intensity physical activity (LPA) or moderate-to-vigorous physical activity (MVPA) on the risk of depression from observational studies, as well as the impact of reallocating time spent in MVPA and LPA to SB.

**Methods:**

Four databases [PubMed, Scopus, SPORTdiscus, and PsycINFO (via EBSCOhost platform)] were searched and analyzed for relevant studies published up to August 2024. Meta-analyses were performed on the estimated regression coefficients (b) and 95% confidence intervals (CIs) for depression symptom scores. All statistical analyses were performed using STATA 16.0.

**Results:**

Twenty-seven studies involving 702,755 participants met the inclusion criteria. Reallocating SB to LPA and MVPA was significantly associated with reductions in depression risk (*b* = −0.04, 95% CI = −0.06 to −0.03, *p* < 0.001; *b* = −0.11, 95% CI = −0.19 to −0.03, *p* = 0.004). Subgroup analyses indicated that reallocating 30 and 60 min of SB to LPA or MVPA was significantly associated with reduced depression risk, with significant differences in PA intensity and age, but not for 10 and 15 min groups. Conversely, reallocating LPA and MVPA to SB was significantly associated with increased depression risk (*b* = 0.11, 95% CI = 0.01 to 0.21, *p* = 0.039; *b* = 0.17, 95% CI = 0.08 to 0.25, *p* < 0.001). Subgroup analyses indicated that reallocating 30 min of LPA or MVPA to SB was significantly associated with increased depression risk, with no difference in PA intensity.

**Conclusions:**

Reallocating SB to PA was beneficial, whereas reallocating PA to SB was detrimental to the risk of depression. The results highlight the importance of considering PA intensity and duration in the development of behavioral guidelines aimed at reducing the risk of depression.

**Systematic review registration:**

https://www.crd.york.ac.uk/PROSPERO/display_record.php?RecordID=546666, identifier: CRD42024546666.

## 1 Introduction

Depression is the second leading contributor to the global disease burden, affecting ~350 million individuals worldwide and resulting in disability for over 44 million people (Vos et al., 2016). A 2017 World Health Organization (WHO) survey further revealed that nearly one billion individuals globally are grappling with various mental health challenges (World Health Organization, 2017). Given the profound societal and economic implications of depression, identifying effective intervention strategies to mitigate and prevent depressive disorders has become a critical area of public health policy.

The 2020 WHO Guidelines emphasize that regular physical activity (PA) among adults aged 18–64 confers substantial health benefits, including improvements in mental health such as reduced symptoms of anxiety and depression, enhanced cognitive function, and better sleep quality—all of which contribute significantly to overall wellbeing (World Health Organization, 2020). In recent years, the relationship between PA and depression risk has garnered considerable academic attention. However, findings regarding the antidepressant effects of PA remain inconsistent. On one hand, a growing body of evidence suggests that PA exerts moderate to significant antidepressant effects, alleviating depressive symptoms in clinical populations (Knapen et al., 2015; Rosenbaum et al., 2014) while also offering protective benefits for non-clinical individuals (Schuch et al., 2018). Even modest amounts of PA, such as walking for 150 min per week, have been associated with a preventive role against depression (Mammen and Faulkner, 2013). These benefits appear to be universal across diverse populations, including pregnant women (Poudevigne and O'Connor, 2006), individuals of different sexes (Chekroud et al., 2018), varying age groups, and residents of different countries (Schuch et al., 2018).

On the other hand, some studies report no significant antidepressant effect of PA, and in certain cases, suggest potential risks to mental health. For instance, a longitudinal study of adolescents found no significant protective effect of physical exercise against depression (Toseeb et al., 2014). Similarly, research on adults has shown no statistically significant association between PA levels and depressive symptoms after controlling for socioeconomic status and other confounding factors (Camacho et al., 1991), with randomized trials reporting analogous findings (Larun et al., 2006). These inconsistencies may stem from selection biases and heterogeneity in the antidepressant effects of PA. Selection bias arises when unmeasured confounders, such as socioeconomic or psychological factors, simultaneously influence both PA engagement and depression risk, potentially skewing estimates. Heterogeneity, meanwhile, reflects variations in PA's antidepressant efficacy across populations with differing demographic and socioeconomic characteristics, underscoring the need for more nuanced investigations into its mechanisms and applicability.

The adverse health consequences of sedentary behavior (time spent sitting) have more recently been identified with the risk of depression, controlling for the influence of moderate to vigorous PA (Zhou et al., 2023). Adverse health consequences of sedentary behavior have been shown for a range of physical and mental health outcomes (Tremblay et al., 2010). Some countries have expanded the scope of their PA guidelines, with joint recommendations on increasing PA and reducing sedentary time (Brown et al., 2013). Time spent standing or being physically active is the time that is not sedentary. Thus, the effect of spending more or less time being either physically active or sedentary will impact outcomes directly. However, those outcomes will also depend, at least in part, on the other activities being displaced. A recent meta-analysis pointed out that although reducing total sedentary time is feasible, in most intervention studies, it has not been clear which time component use the sedentary time has been reallocated (Shrestha et al., 2019). A recent review of isotemporal substitution (IS) studies has demonstrated that the majority of evidence relates to substituting sedentary behaviors (SB) with moderate-to-vigorous physical activity (MVPA), resulting in potential benefits to mortality, general health, mental health, adiposity, fitness, and cardiometabolic biomarkers (Grgic et al., 2018). Although individual studies have reported benefits of replacing sedentary time with physical activity (Rong et al., 2024; Hofman et al., 2022), no prior meta-analysis has quantitatively synthesized these findings to compare the effects of reallocating time to light- versus moderate-to-vigorous-intensity activities or to assess how these associations vary by duration or population characteristics. Our study addresses this gap by integrating evidence across diverse studies to provide generalized estimates.

The application of isotemporal substitution methodology in this systematic review and meta-analysis is grounded in its unique capacity to model the interdependent nature of 24-h time-use behaviors (Mekary et al., 2009; Dorothea et al., 2017). This analytical framework addresses two fundamental aspects of behavioral epidemiology: first, it accounts for the compositional constraints of daily time allocation, recognizing that increased duration in one activity necessarily requires proportional displacement of other activities within the finite 24-h cycle. Second, it provides mathematically robust estimates of health outcomes when examining theoretical time reallocations while maintaining temporal consistency—a critical requirement for developing evidence-based activity guidelines.

Unlike conventional regression approaches, isotemporal substitution analysis specifically examines the health effects of reallocating fixed durations between behavioral domains while holding total time constant. For instance, it can precisely quantify the change in depression risk when 30 min of sedentary behavior is replaced with an equivalent duration of physical activity, controlling for the compositional nature of remaining daily activities. This methodological approach is particularly valuable for investigating mental health outcomes, as it acknowledges both the intensity-dependent effects of substituted activities (Mekary et al., 2009) and the biological plausibility of time-use patterns. Our study employs this framework to elucidate the depressive risk associated with specific activity interchanges, thereby advancing understanding of how behavioral reallocations within constrained temporal resources may influence psychological wellbeing.

The selection of this analytical model was driven by its dual advantages: (a) appropriate handling of the collinear nature of time-use variables through compositional data analysis principles, and (b) generation of clinically interpretable effect estimates for practical behavioral recommendations. These characteristics make isotemporal substitution particularly suited for synthesizing extant research on activity-depression associations while avoiding the ecological fallacies inherent in non-compositional analytical approaches.

## 2 Methods

The systematic review and meta-analysis undertaken in this investigation were meticulously executed in compliance with the Preferred Reporting Items for Systematic Reviews and Meta-Analysis (PRISMA) guidelines (Page et al., 2021). The study protocol for this systematic review was registered in the PROSPERO International Prospective Register of Systematic Reviews (registration number CRD42024546666).

### 2.1 Data sources

Electronic database searches of PubMed, Scopus, SPORTdiscus, and PsycINFO (through the search engine EBSCO) were conducted in August 2024. The last search was performed in August 2024. No limits were imposed on the publication date. The following search terms and keywords were used: (sedentary lifestyle or light PA or sedentary behavior or sedentary time) and (isotemporal substitution or sedentary break or displace sedentary time or replacing or displacing or reallocating or substituting) and (Depression” or “Depressive Symptoms” or “Depressive Symptom” or “Symptom, Depressive” or “Emotional Depression” or “Depression, Emotional). The complete search strategy is shown in Supplementary Tables 1–5. In addition, reference lists were examined to identify studies potentially eligible for inclusion.

### 2.2 Eligibility criteria

**Figure 1:**
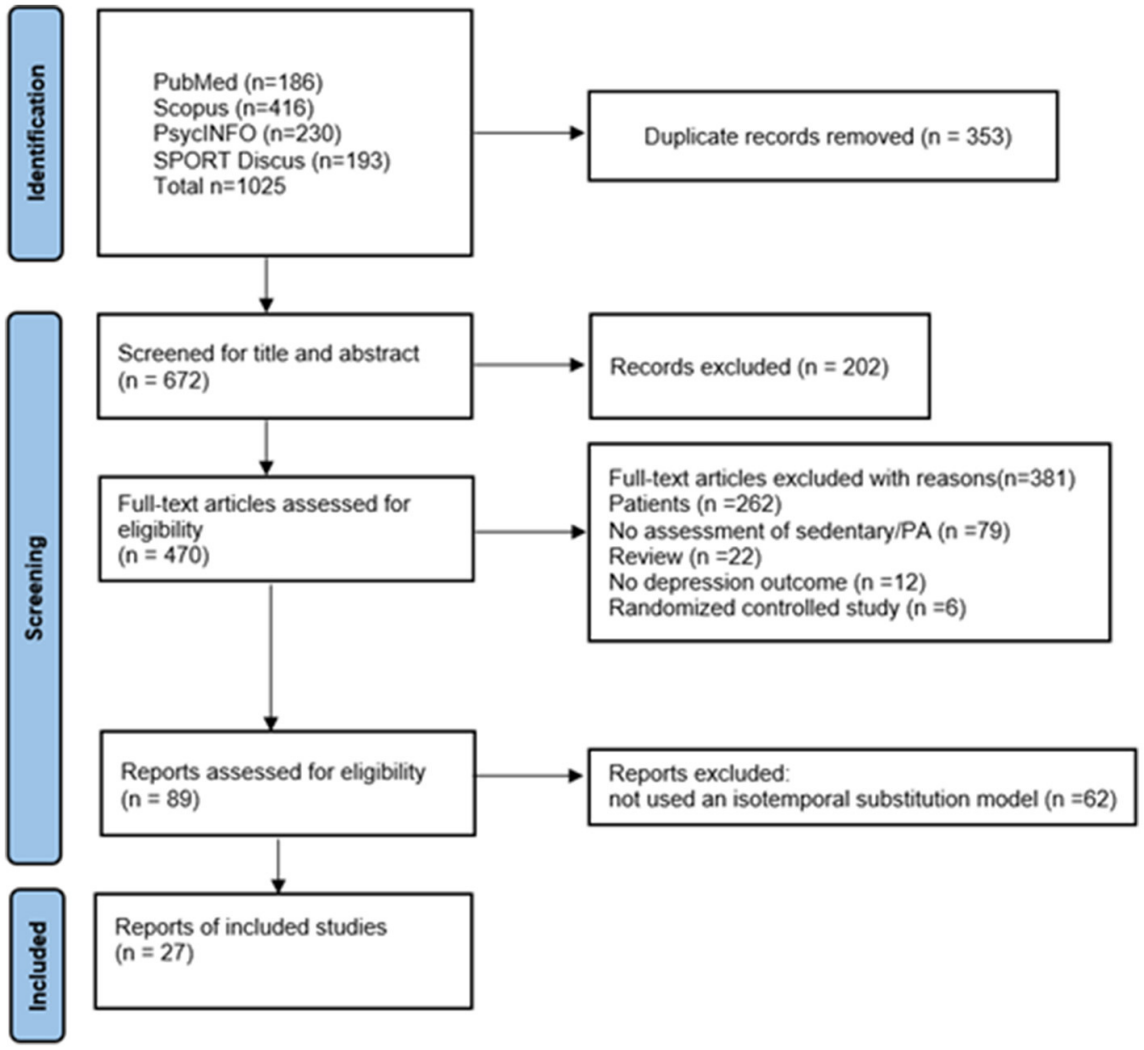
PRISMA diagram.

The review encompassed studies that adhered to the following criteria: (1) they were designed as observational cohort, cross-sectional, or case-control studies; (2) they provided objective or self-reported measures of physical activity (PA) and sedentary behavior (SB); (3) they utilized isotemporal substitution models to report the impact of substituting SB with light-intensity physical activity (LIPA) or moderate-to-vigorous physical activity (MVPA) on depression symptoms or major depressive disorder as the primary outcomes of interest; (4) they targeted general populations without focusing on specific disease groups; and (5) they presented original research findings. The screening of titles, abstracts, and full texts was independently performed by two reviewers (YW and JS) using EndNote X9 for reference management and duplicate removal. Any discrepancies between reviewers were resolved through discussion or by consulting a third reviewer (SL) when consensus could not be reached. The study selection process, including the number of records excluded at each stage, is summarized in the PRISMA flow diagram (Figure 1). Eligibility for inclusion was confirmed before proceeding to a full-text review, after which data extraction was performed.

### 2.3 Data collection

The following information was extracted: the sample characteristics, objective or self-report measuring method of physical activity, quantity of inactive or screen or sedentary time being replaced, outcomes of interest, analytical approach, and main conclusions from each treatment are discussed. In addition, data that would assist in findings from the meta-analysis of the different studies (i.e., regression coefficient and 95% CI representing the effect of replacing SB with more active behavior on the outcome of interest) were extracted. To maximize the generalizability of the findings, the original authors were contacted when data were unavailable.

### 2.4 Risk of bias

The quality of each study was assessed using an adjusted version of Newcastle Ottawa (Wells et al., 2000). This scale contains eight items categorized into three domains (selection, comparability, and exposure). A star system is used to enable semi-quantitative assessment of study quality such that the highest-quality studies are awarded a maximum of one star per item, except for the comparability domain, which allows the allocation of two stars. Thus, the score ranges from zero to nine stars (minimum scores for cohort and cross-sectional studies were seven and five, respectively).

### 2.5 Data synthesis and analytical approach

A one-step, individual participant data meta-analysis was performed. All analyses were performed using STATA software (version 16.0). Estimated regression coefficients (b) and 95% CIs were combined and used in the meta-analysis to assess the risk of depression. The random effects model (Hunter-Schmidt approach) was used to summarize the pooled b values in all cases. In this case, the range of values was reported. The likelihood approach with random effects was used to better account for the imprecision in the estimate of between-study variance (Hardy and Thompson, 1996). When studies presented several statistical risk-adjustment models, only values associated with the statistical models containing the fewest additional covariates were considered to improve comparability across studies.

The percentage of total variation across the studies due to heterogeneity (Cochran's Q-statistic) was estimated using *I*^2^. Values *I*^2^ of < 25%, 25%, 50%, and >50% were considered small, medium, and large amounts of heterogeneity, respectively (Higgins and Thompson, 2002). Small-study effects bias was assessed using the extended Egger's test, funnel plots were used to investigate publication bias among studies graphically (Egger et al., 1997; Higgins et al., 2019), and a sensitivity analysis was conducted to assess the robustness of the summary estimates to determine whether a particular study accounted for the heterogeneity. Therefore, a series of analyses were conducted by sequentially omitting one study at each turn.

## 3 Results

### 3.1 Study selection

The search strategy initially identified 1,025 articles (Figure 1). Eighty-nine articles were retrieved after the initial screening. Of these, 62 were rejected (they did not use an isotemporal substitution model and had no study outcomes). Finally, 27 studies were included in this systematic review and meta-analysis. Exclusion criteria were: (a) clinical populations, (b) non-observational designs, (c) absence of isotemporal substitution analysis, and (d) no depression outcomes.

### 3.2 Study characteristics

Twenty-seven studies included a total of 702,755 participants. The sample size ranged from 139 individuals to 360,047 individuals. Sex was evenly distributed (women, 56.95%), and the mean age was 50.46 years. Eleven of the studies were cohort studies (Chiba et al., 2022; Monteagudo et al., 2023; Zhu et al., 2024; Rong et al., 2024; Hallgren et al., 2020; Hofman et al., 2022; Cao et al., 2022; Cabanas-Sánchez et al., 2021; Mekary et al., 2013; Sampasa-Kanyinga et al., 2021; Kandola et al., 2022), observational investigations, and 16 of the studies were cross-sectional studies (Araki et al., 2022; Curtis et al., 2023; Park et al., 2024; Nam et al., 2023; Dillon et al., 2018; Liu et al., 2023; Yasunaga et al., 2018; Gilchrist et al., 2021; Sadarangani et al., 2023; Rethorst et al., 2017; Meneguci et al., 2024; Wei et al., 2019; Tully et al., 2020; Zhou et al., 2024; de Faria et al., 2022; Tang et al., 2024). The characteristics of these studies are summarized in Supplementary Table S5. The methodological quality of the included studies is presented in Supplementary Table S6. In total, 24 of the 27 studies were of high quality. Only one study was reported to be of low quality.

### 3.3 Effects of reallocating sedentary time to physical activity

**Figure 2:**
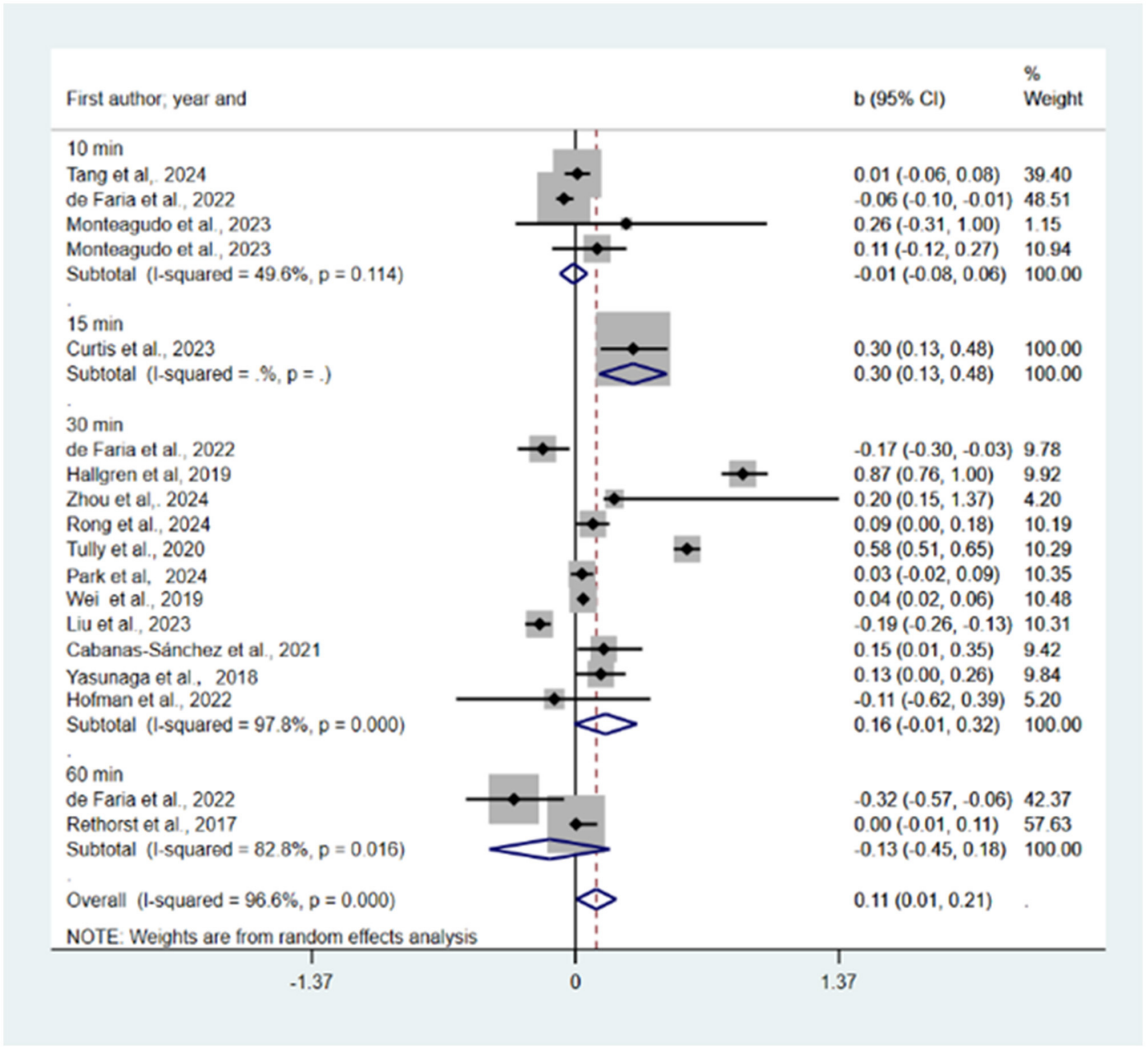
Pooled effect size of substituting SB by LPA on the risk of depression.

Reallocation of 10, 15, 30, 60 min and all sedentary time to LPA was predicted to be associated with reductions in depression (*b* = −0.05, 95% CI = −0.09, −0.02, *p* = 0.001; *b* = −0.14, 95% CI = −0.41, 0.14, *p* = 0.325; *b* = −0.04, 95% CI = −0.08, −0.01, *p* = 0.008; *b* = −0.04, 95% CI = −0.07, 0.02, *p* = 0.001; *b* = −0.04, 95% CI = −0.06, −0.03, *p* = 0.000; see Figure 2). One study (Cabanas-Sánchez et al., 2021) was excluded from the sensitivity analysis. There was a statistically significant publication bias in the funnel plot (see Supplementary Figure 1) and Egger's test (*P* = 0.002).

**Figure 3:**
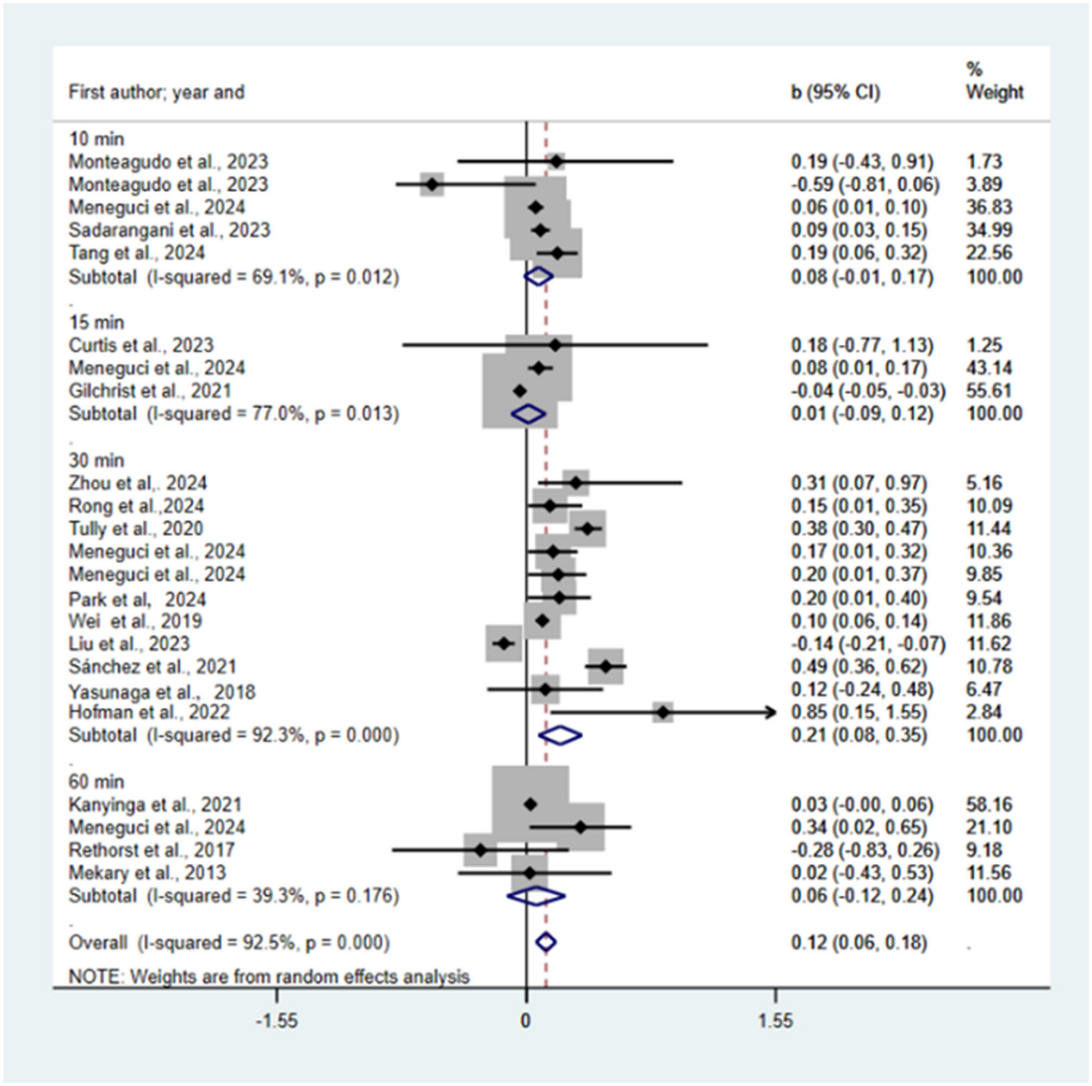
Pooled effect size of substituting SB by MVPA on risk of depression.

Reallocation of 10, 15, 30, 60 min and all sedentary time to MVPA was predicted to be associated with reductions in depression (*b* = −0.08, 95% CI = −0.19, 0.02, *p* = 0.118; b = −0.03, 95% CI = −0.20, 0.13, *p* = 0.692; *b* = −0.13, 95% CI = −0.22, −0.05, *p* = 0.003; *b* = −0.17, 95% CI = −0.31, 0.03, *p* = 0.021; *b* = −0.11, 95% CI = −0.19, −0.03, *p* = 0.004; see Figure 3). Two studies (Gilchrist et al., 2021; Liu et al., 2023) were excluded from sensitivity analysis. We found no statistically significant publication bias using a funnel plot (see Supplementary Figure 2) and Egger's test (*P* = 0.91).

### 3.4 Effects of reallocating physical activity to sedentary time

**Figure 4:**
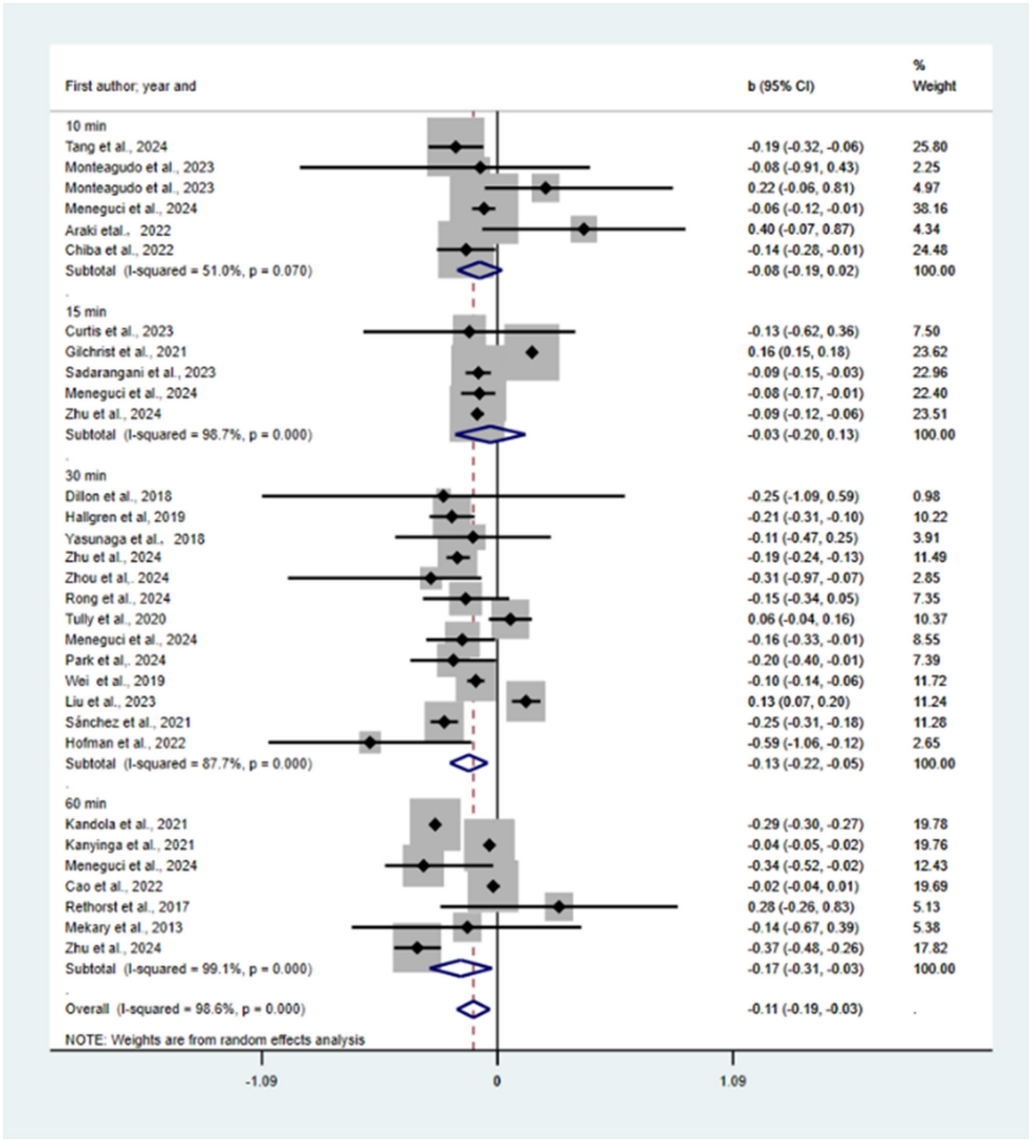
Pooled effect size of substituting LPA by SB on the risk of depression.

Reallocation of 10, 15, 30, 60 min and all LPA time to sedentary time was predicted to be associated with reductions or increases in risk of depression (*b* = −0.01, 95% CI = −0.08, 0.06, *p* = 0.787; *b* = 0.30, 95% CI = 0.13, 0.48, *p* = 0.001; *b* = 0.16, 95% CI = −0.01, 0.32, *p* = 0.058; *b* = −0.13, 95% CI = −0.45, 0.18, *p* = 0.402; *b* = 0.11, 95% CI = 0.01, 0.21, *p* = 0.039; see Figure 4). We found no statistically significant publication bias using funnel plot (see Supplementary Figure 3) and Egger's test (*P* = 0.38).

**Figure 5:**
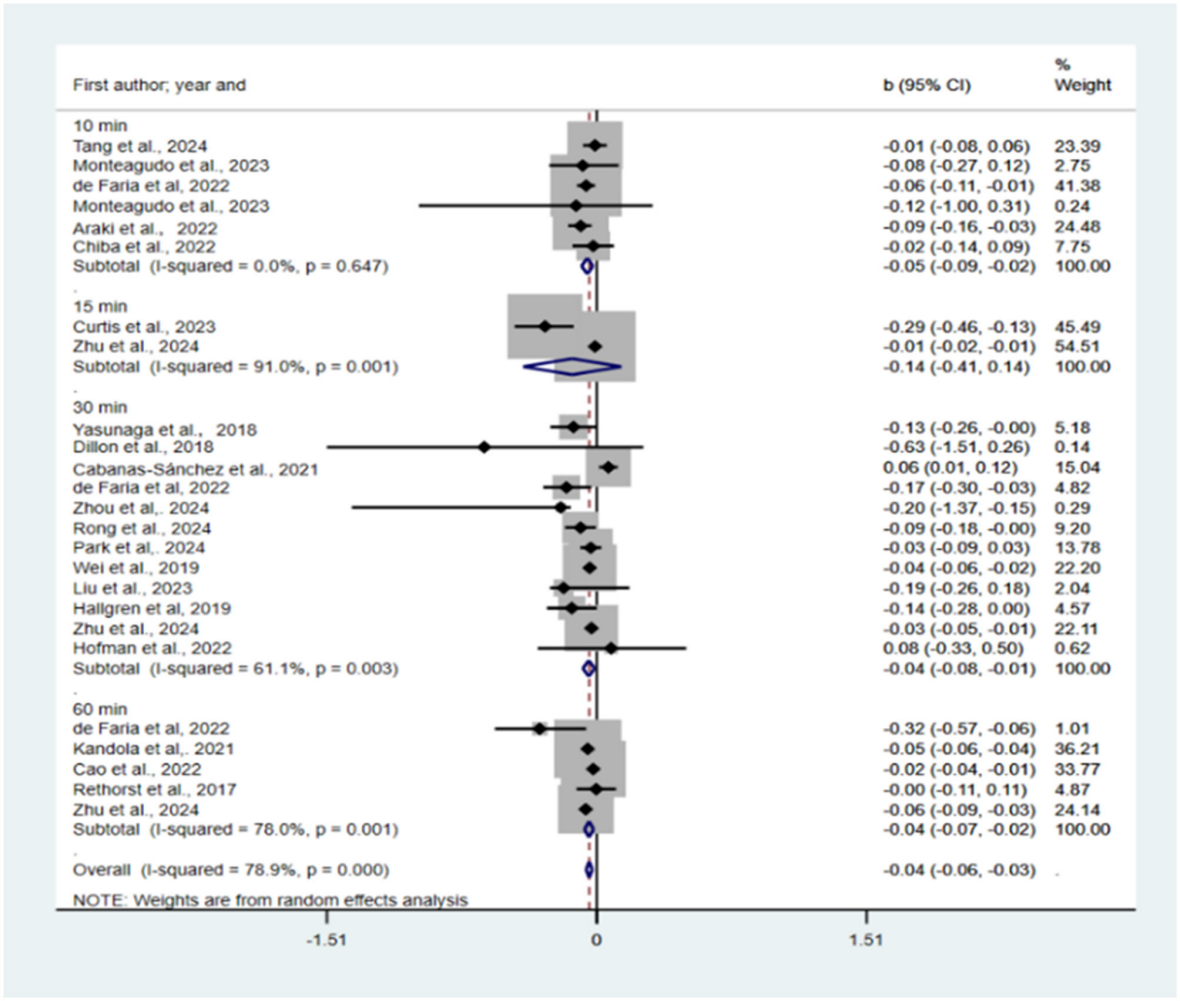
Pooled effect size of substituting MVPA by SB on the risk of depression.

Reallocation of 10, 15, 30, 60 min and all MVPA time to sedentary time was predicted to be associated with increases in risk of depression (*b* = 0.08, 95% CI = −0.01, 0.17, *p* = 0.098; *b* = 0.01, 95% CI = −0.09, 0.12, *p* = 0.809; *b* = 0.28, 95% CI = 0.09, 0.47, *p* = 0.002; *b* = 0.06, 95% CI = −0.12, 0.24, *p* = 0.499; *b* = 0.17, 95% CI = 0.08, 0.25, *p* = 0.000; see Figure 5). One study (Hallgren et al., 2020) was excluded from sensitivity analysis. We found no statistically significant publication bias using a funnel plot (see Supplementary Figure 4) and Egger's test (*P* = 0.38).

### 3.5 Subgroup analysis

Table 1 shows the results of the subgroup analyses that examined the pooled results' stability and explored the potential sources of heterogeneity. Regarding the source of heterogeneity in reallocating Sedentary Time to LPA, significant between-group heterogeneity was found for study design, age, and duration of sedentary time, indicating that these factors may affect the pooled ORs. Significant within-subgroup heterogeneity in the studies on MVPA was found in subgroups such as Cohort, Cross-sectional, Adult, and Instrumental measures of PA, 15, 60, indicating that the pooled ORs in these groups may influence overall heterogeneity. Regarding reallocating Sedentary Time to MVPA, significant between-group heterogeneity was found for age, depression scales, and PA measurements, indicating that these factors may affect the pooled ORs. Significant within-group heterogeneity was also found in most subgroups except for adolescents and adults, with 10 min and 15 min durations. Additionally, subgroup analyses of different intensities revealed significant differences in the effect sizes between replacing SB with LPA and MVPA at 15 min, 30 min, 60 min, and all times, but not for 10 min.

**Table 1:** Results of the subgroup analysis by meta-regression for replacing sedentary time with LPA, MVPA.

**Subgroup**			**Replacing sedentary time with LPA**					**Replacing sedentary time with MVPA**			
		***N***	**OR (95% CI)**	*I*^2^ **(%)**	**p** ^a^	**P** ^b^	***N***	**OR (95% CI)**	*I*^2^ **(%)**	**p** ^a^	**P** ^b^
All reports		24	−0.04 (−0.06, −0.03)	67.44	0.00	-	29	−0.14 (−0.19, −0.08) ^**^	95.34	0.00	-
Study design	Cohort	11	−0.03 (−0.05, −0.02)	74.28	0.00	0.02	13	−0.15 (−0.23, −0.06)	97.58	0.00	0.63
	Cross-sectional	13	−0.07 (−0.11, −0.04)	38.13	0.01		16	−0.12 (−0.17, −0.08)	63.77	0.00	
Age	Adolescent	6	−0.07 (−0.12, −0.02)	24.73	0.12	0.01	4	−0.05 (−0.08, −0.01)	3.77	0.07	0.00
	Adult	7	−0.05 (−0.07, −0.03)	6.73	0.07		7	−0.29 (−0.31, −0.26)	2.06	0.15	
	Older	4	−0.05 (−0.06, 0.03)	0	0.27		10	−0.11 (−0.17, −0.05)	72.15	0.00	
	Mixed	7	−0.02 (−0.03, −0.01)	39.47	0.00		8	−0.11 (−0.17, −0.06)	78.93	0.00	
Depression scales	CES	6	−0.02 (−0.08, 0.03)	0	0.45	0.21	6	−0.06 (−0.11, −0.02)	6.21	0.02	0.02
	GDS	3	−0.08 (−0.13, 0.03)	0	0.42		8	−0.14 (−0.21, −0.06)	67.41	0.00	
	PHQ	3	−0.05 (−0.06, −0.04)	0	0.55		3	−0.20 (−0.28, −0.12)	87.51	0.00	
	Others	12	−0.04 (−0.05, −0.02)	68.13	0.00		12	−0.12 (−0.17, −0.06)	81.37	0.00	
PA measures	Self-report	6	−0.03 (−0.05, −0.02)	25.05	0.10	0.24	12	−0.08 (−0.11, −0.05)	61.09	0.00	0.01
	Instrumental	18	−0.05 (−0.07, −0.03)	69.85	0.00		17	−0.17 (−0.23, −0.11)	85.40	0.00	
TIME	10	6	−0.05 (−0.09, −0.02)	0	0.65	0.03	6	−0.09 (−0.17, −0.01)	26.68	0.07	0.14
	15	2	−0.01 (−0.03, 0.00)	1.97	0.00		4	−0.09 (−0.12 0.07) ^**^	0.00	0.99	
	30	11	−0.05 (−0.06, −0.03)	14.03	0.19		12	−0.16 (−0.21 −0.10) ^**^	64.33	0.00	
	60	5	−0.04 (−0.06, −0.02)	61.16	0.00		7	−0.17 (−0.29 −0.06) ^**^	98.54	0.00	

CS, Cross-sectional study; SES, socioeconomic status; P^a^ and P^b^, heterogeneity within and between subgroups, respectively. ^**^Significant differences in the effect sizes between replacing sedentary time with LPA and MVPA.

As for the source of heterogeneity in reallocating Sedentary Time to LPA, Table 2 shows significant between-group heterogeneity for age and duration of sedentary time, indicating that these factors may affect the pooled ORs. Significant within-subgroup heterogeneity in the studies on MVPA was found in subgroups such as Cross-sectional, Adolescent, Adult, CES, Self-report, instrumental, 30 min, and 60 min, indicating that the pooled ORs in these groups may influence overall heterogeneity. Regarding the reallocation of Sedentary Time to MVPA, significant between-group heterogeneity was found for all subgroups, indicating that these factors may affect the pooled ORs. Significant within-group heterogeneity was also found in most subgroups, except for age, PHQ, and 60 min. In general, the pooled ORs for reallocating Sedentary Time and MVPA to each other were inconsistent in most analyses. The high heterogeneity among the included studies may be attributed to the large number of included studies and disparities in the study design, depression ascertainment, and duration of ST. Additionally, subgroup analyses of different intensities revealed significant differences in the effect sizes between replacing LPA and MVPA with SB only at 15 min.

**Table 2:** Results of the subgroup analysis by meta-regression for replacing LPA, MVPA with sedentary time.

**Subgroup**			**Replacing LPA with sedentary time**					**Replacing MVPA with sedentary time**			
		***N***	**OR (95% CI)**	*I*^2^ **(%)**	**p** ^a^	**P** ^b^	***N***	**OR (95% CI)**	*I*^2^ **(%)**	**p** ^a^	**P** ^b^
All reports		18	0.11 (0.01, 0.21)	75.32	0.00	-	23	0.12 (0.06, 0.18)	83.64	0.00	-
Study design	Cohort	5	0.04 (−0.01, 0.09)	0.00	0.40	0.15	7	0.03 (−0.05, 0.12)	12.53	0.01	0.05
	Cross-sectional	13	0.12 (0.02, 0.23)	96.20	0.00		16	0.13 (0.08, 0.18)	84.14	0.00	
Age	Adolescent	6	−0.05 (−0.12, 0.02)	47.67	0.01	0.00	5	0.00 (−0.04, 0.04)	57.14	0.00	0.00
	Adult	8	0.12 (−0.06, 0.31)	83.48	0.00		9	0.17 (−0.02, 0.37)	91.14	0.00	
	Older	3	0.04 (0.02, 0.07)	1.29	0.19		7	0.09 (0.06, 0.12)	0.00	0.34	
	Mix	1	0.87 (0.75, 0.99)	-	-		2	0.11 (−0.00, 0.21)	3.36	0.03	
Depression scales	CES	5	−0.05 (−0.15, 0.04)	74.77	0.00	0.03	6	−0.01 (−0.05, 0.02)	62.42	0.00	0.00
	GDS	2	0.14 (0.03, 0.24)	0.00	0.89		8	0.18 (0.08, 0.28)	75.70	0.00	
	PHQ	2	0.04 (0.02, 0.06)	0.00	0.85		2	0.10 (0.06, 0.14)	0.00	0.31	
	Others	9	0.19 (−0.03, 0.41)	97.41	0.00		7	0.12 (−0.03, 0.27)	76.36	0.00	
PA measures	Self-report	5	0.15 (0.02, 0.27)	95.30	0.00	0.50	14	0.05 (0.02, 0.09)	77.86	0.00	0.03
	Instrumental	13	0.08 (−0.05, 0.22)	94.51	0.00		9	0.23 (0.08, 0.39)	65.98	0.00	
TIME	10	4	−0.02 (−0.07, 0.03)	23.52	0.11	0.01	5	0.08 (0.01, 0.15)	52.54	0.01	0.00
	15	1	0.30 (0.12, 0.47)	-	-		3	−0.03 (−0.05, 0.00) ^**^	8.37	0.01	
	30	11	0.16 (−0.01, 0.32)	97.90	0.00		11	0.21 (0.09, 0.32)	88.87	0.00	
	60	2	−0.13 (−0.45, 0.18)	28.70	0.02		4	0.03 (−0.01, 0.07)	1.02	0.18	

CS, Cross-sectional study; SES, socioeconomic status; P^a^ and P^b^, heterogeneity within and between subgroups, respectively. ^**^ Significant differences in the effect sizes between replacing LPA and MVPA with sedentary time.

## 4 Discussion

### 4.1 Main finding

To the best of our knowledge, this is the first meta-analysis to quantitatively examine the association between substitutions of PA and SB with each other and depression risk. This meta-analysis included 27 observational studies with 702,755 participants and found that equal time exchange of SB with PA may lead to additional reductions in the risk of depression. In contrast, equal time exchange of PA with SB may lead to additional increases in the risk of depression. However, some results were inconsistent when a subgroup analysis was conducted. Meanwhile, we also found some sources of the overall heterogeneity through subgroup analysis and excluded four studies' data through sensitivity analysis. Funnel plot analysis also confirmed that some research results may be subject to publication bias.

The main findings of this meta-analysis showed that reallocating sedentary time and PA to each other may result in reductions or increases in the risk of depression. These findings suggest the potential benefits of replacing SB with PA. This might provide alternative intervention strategies, as it may be more feasible and less challenging than more strenuous activity (which is also more difficult than lighter activity to fit into daily life routines, especially in particular domains, such as work or education, where SB is particularly prevalent), to enhance mental health among the general population (Shrestha et al., 2019). In addition, LPA and MVPA could be feasible strategies to increase the total volume of PA among those considered already active and, therefore, could bring additional mental benefits (Kokkinos, 2012). However, the current evidence is not consistent for reallocating 15 min of SB to LPA based on a non-significant *p*-value. Possible reasons include an insufficient number of studies included in this time group, and another possible reason is that the protective effect against depression may be smaller for shorter substitution durations compared to longer durations. Additionally, this study found evidence of publication bias in the overall effect estimate when using LPA to replace SB, and these results should be interpreted with caution.

### 4.2 Comparison with existing evidence

The results of our study suggest that LPA and MVPA are both significantly associated with lower odds of depression. This finding aligns with previous meta-analyses employing non-isotemporal substitution models, demonstrating a consistent inverse association between PA and incident depression risk (Dishman et al., 2021; Schuch et al., 2018). However, the effect sizes differ significantly between replacing SB with LPA and MVPA at 15 min, 30 min, 60 min, and all times, but not for 10 min (as shown in Table 1). This dose-response relationship is consistent with previous findings from non-isotemporal substitution meta-analyses, demonstrating an inverse association between varying doses of PA and lower risk of incident depression (Pearce et al., 2022). Another recent dose-response meta-analysis also confirmed that the risk of incident depression was reduced by 3% (RR: 0.97, 95% CI: 0.95–0.98) for every 5 MET-h/week increase in LTPA < 25 MET-h/week (LTPA: leisure time PA; Guo et al., 2022). This reinforces the significance of the intensity and duration of PA, which has also been demonstrated in previous studies (Singh et al., 2023; Mekary et al., 2013). Specifically, Mekary et al. (2013) reported that a protective effect against depression was associated with substituting 60 min of television viewing with a brisk or very brisk walk, whereas an easy-paced walk did not exhibit the same association. A recent systematic review of the literature has further substantiated that MVPA is linked to more pronounced improvements in depressive symptoms relative to LPA (Singh et al., 2023). The observed disparity in association with the intensity of PA may be due to the inadequacy of LPA in inducing the essential hormonal changes essential for preventing depression (Goldfarb et al., 1998). In summary, we may assume that there is a dose-response relationship between reallocating sedentary time to PA and the risk of depression.

Although some studies have indicated that LPA mitigates the severity of depression, thereby highlighting its significance (Yasunaga et al., 2018; Dillon et al., 2018), these studies primarily focused on middle-aged and elderly populations. A cohort study encompassing individuals aged 18–74 years, which is analogous to the demographics of our subjects, did not identify a significant association between LPA and depressive symptoms (Rethorst et al., 2017). Cao et al. (2022) also observed that replacing SB or sleep with walking could lead to a reduction in anxiety symptoms, with the association potentially moderated by age-related factors. Our meta-analysis study separated MPA and LPA, finding that reallocating time from SB to MPA was associated with lower depression symptoms, while it was also observed with LPA. Based on these conflicting results, it is imperative to conduct additional research on various age groups and levels of PA intensity. MVPA has been shown to confer greater health benefits than LPA when displacing an equivalent amount of SB, resulting in an estimated 40% and 20% reduction in the risk of all-cause mortality, respectively—LPA may still present a more viable and pertinent alternative for physical engagement in scenarios where MVPA is not feasible. This includes office settings where MVPA might be impractical, individuals who are physically limited and unable to partake in MVPA, such as the elderly or those with frailty, and populations that could experience health benefits from increased PA, regardless of the intensity level.

### 4.3 Subgroup analysis

The findings of this study further elucidate the potential adverse effects associated with reallocating PA to SB. Despite many studies reporting non-significant outcomes, a general consensus has emerged regarding the effects of substituting SB for 30 min with either LPA or MVPA, demonstrating significant benefits. Subgroup analyses revealed significant heterogeneity across studies attributable to differences in methodologies, age groups, measurement techniques, and intervention durations. Notably, age emerged as a significant factor in all four substitution models. In addition, no significant differences were observed between the effects of MVPA and LPA replacement with SB. Between-group heterogeneity assessments indicated considerable variability within most subgroups of the four models examined in this study, likely because of the large number of literature sources and the many subgroup classifications applied. Heterogeneity may also stem from inter-group differences, as discussed in the subgroup heterogeneity tests, with age differences significantly affecting study outcomes. In summary, these heterogeneous findings, marked by considerable variability, require careful interpretation.

However, certain subgroups within this study, characterized by large sample sizes and low heterogeneity, were identified. For instance, within the PA substitution for the SB model, subgroups involving adolescents and adults, the CES Depression Scale, and the 10-min substitution subgroup showed non-significant heterogeneity. Similarly, within the SB substitution for the PA model, subgroups such as cohort studies and elderly populations exhibited a high number of studies with low heterogeneity. In brief, Replacing SB with PA has the strongest protective effect against depression in older adults, while adolescents show no significant impact, suggesting age-specific intervention strategies. Replacing MVPA with SB worsens depression risk more than replacing LPA, emphasizing the importance of preserving higher-intensity movement. Short sedentary breaks (< 30 min) show minimal harm, but prolonged sitting—especially displacing MVPA—has clearer negative effects, supporting frequent activity interruptions for mental health.

Another important finding in our meta-analysis related to the reallocation of time between SB and LPA is the asymmetry of the results. Replacing SB with LPA and MVPA significantly reduced the risk of depression in different time subgroups, with significant differences in intensity. However, replacing LPA and MVPA with SB significantly increased the risk of depression only in the 30-min alternate time subgroup, and no differences in intensity were observed. These asymmetry patterns were observed for all substitution subgroups between SB and LPA, independent of the time involved. These asymmetric relationships are consistent with previous studies on compositional isotemporal substitution (Chastin et al., 2015b; Biddle et al., 2018). de Faria et al. (2022) considered that the detrimental effects of time reduction in LPA are greater than the estimated beneficial results that occur from its increase. Another possible explanation for this fact relies on the time distribution among the movement behaviors within 24 h (Chastin et al., 2015a). Therefore, any reallocation from the LPA constitutes a substantial proportion of its time, which is not true for SB. Together, these results reinforce the need for interventions that at least promote the maintenance of LPA levels to reduce depression. Considering our sample, in addition to the above factors, the asymmetry may also be due to insufficient literature for replacing LPA and MVPA with SB-related subgroups.

### 4.4 Potential mechanisms

Several potential mechanisms may elucidate the beneficial associations observed through isotemporal substitution analyses, posing that replacing sedentary time with PA could exert antidepressant effects. First, the interchangeability of sedentary time with PA can influence depression through a spectrum of biological and psychosocial pathways. These include the reduction of inflammatory markers, modulation of neuroplasticity, and enhancement of self-esteem, which are known to contribute to mental wellbeing (Wheeler et al., 2020). Second, contemporary research has highlighted the advantages of interrupting prolonged periods of SB with bouts of PA in adults. Such interruptions have been suggested to confer cognitive benefits, including promoting brain plasticity, alleviating fatigue, and ameliorating cognitive performance (Bojsen-Møller et al., 2020; Filiou and Sandi, 2019). Furthermore, avoiding extended sedentary periods may help sustain a steady level of mitochondrial activity. This could mitigate the risk of mitochondrial dysfunction in the brain, a condition implicated in the pathophysiology of depression (Molendijk et al., 2018). In summary, the isotemporal substitution of sedentary time with PA presents a multifaceted approach to potentially reducing the risk of depression, supported by a convergence of biological, psychological, and cognitive evidence. These insights underscore the need for further research to elucidate the intricate relationship between PA, SB, and mental health.

### 4.5 Strengths and limitations

The strengths of this study include its longitudinal design (ten cohort studies), adequate sample size (702,755), and the use of accelerometry to objectively measure PA and SB (14 studies with instrumental measures). Therefore, although self-reported measures provide rich contextual information on activity mode (i.e., sports practice, transportation, or play) or domain (i.e., family-based or school-based), they typically overestimate youth PA levels compared with objective measures and are generally unable to accurately classify PA intensity (Fairclough and Noonan, 2020). A key strength of this study lies in its rigorous application of isotemporal substitution modeling, which provides a theoretically grounded framework to quantify the independent health effects of displacing sedentary time with PA of varying intensities. This methodology enables precise estimation of depression risk by accounting for the constrained nature of time allocation while controlling for total activity volume, thereby offering clinically meaningful insights for behavioral intervention design.

This meta-analysis had limitations at the level of the individual studies examined and at the level of the review that was feasible with the data available. The cross-sectional nature of the pooled observations does not allow definitive conclusions to be drawn regarding the causal relationship between the variables of interest. Second, isotemporal substitution modeling has some limitations. The principle underpinning isotemporal substitution modeling and multiple regression analysis brings some issues, such as not contemplating the codependent nature of PA data, which may further limit its utility in this field (Batacan et al., 2015). The isotemporal substitution model provides useful insights into activity trade-offs but assumes linear, independent effects of time reallocation, which may oversimplify real-world behavioral interactions. These limitations could lead to overestimated effect sizes, particularly for MVPA substitution, since the model doesn't fully account for compensatory behaviors or threshold effects in depression risk. Finally, to increase comparability among studies, only effect sizes associated with less adjusted models were combined and analyzed, which could additionally limit the validity of the reported results. To further increase comparability among studies, the estimated b and 95% CIs of studies using 10-min blocks as units of exchange were scaled up to 30 min, so all included studies would consistently show the effects of replacing 30 min of sedentary time with LIPA and MVPA on the selected outcome. Although these estimations conformed to the linearity properties of the method used (i.e., linear regression analysis), the results should be interpreted cautiously.

### 4.6 Clinical implications and future research

Our study has important implications for public mental health. Depression is a common and burdensome disorder, with a median age of onset in the early 20s (Kessler and Bromet, 2013). It is projected to be a leading cause of disease burden in high-income countries and the second cause of disease burden worldwide by 2030 (Mathers and Loncar, 2006). Additionally, SB is prevalent and pervasive in modern society and is a risk factor that can be avoided by changing one's personal lifestyle. With the health benefits of PA, many countries are paying more attention to PA promotion while ignoring a series of health problems due to SB (Piercy and Troiano, 2018; Weggemans et al., 2018). Given the association between sedentary lifestyle and mental health disorders observed in the present and other studies, public health campaigns to reduce the risk of mental disorders should advocate regular PA and reduce SB (Zhou et al., 2024). The findings of this meta-analysis highlight the importance of considering the combined effects of movement and non-movement behaviors on the risk of depression (i.e., replacing sedentary time with MVPA predicts a stronger association than LPA). Recently, a 24-h analysis approach has been suggested to evaluate the codependent nature of the daily proportion of movement and non-movement behaviors on health, and a novel analytical approach has been proposed (i.e., compositional analysis) to account for it (Batacan et al., 2015). Therefore, this new approach can be used on epidemiological observational and experimental data to provide new insights into the relationship between PA and health and develop a new 24-h PA guideline based on compositional analysis (Tremblay et al., 2016).

To enhance real-world applicability, public health guidelines should prioritize integrating objective activity monitoring (e.g., wearables) with contextual self-reports to better tailor interventions for different settings like schools or communities. Future research should address the model's inability to capture compensatory behaviors after activity substitution, while also exploring how sociocultural factors influence the relationship between SB and mental health across diverse populations. To address current methodological gaps for adapting 24-h movement healthy lifestyle, future studies also should employ hybrid assessment protocols combining accelerometry with ecological momentary assessment (EMA) to simultaneously capture objective activity patterns and real-time contextual factors influencing compensatory behaviors. Additionally, culturally adapted intervention trials are needed to examine how socioeconomic status and built environment modify the SB-mental health relationship across global regions, particularly in low- and middle-income countries underrepresented in current evidence.

## 5 Conclusions

Reallocation of SB to PA may be beneficial, and reallocation of PA to SB may be detrimental to the risk of depression. However, when SB was replaced with MVPA, the predicted impacts were stronger and more apparent. These findings also point to the potential benefits of replacing SB with LPA, which may benefit those less able to tolerate or accommodate higher-intensity activities, including many older adults. Future research should move beyond observational evidence and identify more robust indications of depression outcomes by experimentally reallocating time spent in SB with physical activities of different intensities.

## Data Availability

The original contributions presented in the study are included in the article/Supplementary material, further inquiries can be directed to the corresponding author.

## References

[B1] Araki K. Yasunaga A. Shibata A. Hattori K. Honma R. Sato N. . (2022). Cross-sectional associations between replacing sedentary behavior with physical activity by accelerometer-measured and depression in frail older adults: an isotemporal substitution approach. Japanese J. Phys. Fit. Sports Med. 71, 185–192. 10.7600/jspfsm.71.185

[B2] Batacan R. Duncan M. Dalbo V. Tucker P. Fenning A. (2015). Effects of light intensity activity on CVD risk factors: a systematic review of intervention studies. BioMed Res. Int. 2015:596367. 10.1155/2015/596367 26543862 PMC4620294

[B3] Biddle G. G. Edwardson C. Henson J. Davies M. Khunti K. . (2018). Associations of physical behaviours and behavioural reallocations with markers of metabolic health: a compositional data analysis. Int. J. Environ. Res. Public Health 15:2280. 10.3390/ijerph15102280 30336601 PMC6210541

[B4] Bojsen-Møller E. Ekblom M. Tarassova O. Dunstan D. Ekblom O. (2020). The effect of breaking up prolonged sitting on paired associative stimulation-induced plasticity. Exp. Brain Res. 238, 2497–2506. 10.1007/s00221-020-05866-z 32860117 PMC7541377

[B5] Brown W. J. Bauman A. E. Bull F. Burton N. W. (2013). Development of Evidence-Based Physical Activity Recommendations for Adults (18-64 Years). Report prepared for the Australian Government Department of Health. Australia: Commonwealth of Australia.

[B6] Cabanas-Sánchez V. Esteban-Cornejo I. García-Esquinas E. Ortolá R. Ara I. Rodríguez-Gómez I. . (2021). Cross-sectional and prospective associations of sleep, sedentary and active behaviors with mental health in older people: a compositional data analysis from the Seniors-ENRICA-2 study. Int. J. Behav. Nutr. Phys. Act. 18:124. 10.1186/s12966-021-01194-9 34530862 PMC8444566

[B7] Camacho T. C. Roberts R. E. Lazarus N. B. Kaplan G. A. Cohen R. D. (1991). Physical activity and depression: evidence from the Alameda County study. Am. J. Epidemiol. 134, 220–231. 10.1093/oxfordjournals.aje.a116074 1862805

[B8] Cao Z. Xu C. Zhang P. Wang Y. (2022). Associations of sedentary time and physical activity with adverse health conditions: outcome-wide analyses using isotemporal substitution model. EClinicalMedicine 48:101424. 10.1016/j.eclinm.2022.101424 35516443 PMC9065298

[B9] Chastin S. Palarea-Albaladejo J. Dontje M. Skelton D. (2015a). Combined effects of time spent in physical activity, sedentary behaviors and sleep on obesity and cardio-metabolic health markers: a novel compositional data analysis approach. PLoS ONE 10:e0139984. 10.1371/journal.pone.0139984 26461112 PMC4604082

[B10] Chastin S. F. Egerton T. Leask C. Stamatakis E. (2015b). Meta-analysis of the relationship between breaks in sedentary behavior and cardiometabolic health. Obesity 23, 1800–1810. 10.1002/oby.21180 26308477

[B11] Chekroud S. R. Gueorguieva R. Zheutlin A. B. Paulus M. Krumholz H. M. Krystal J. H. . (2018). Association between physical exercise and mental health in 1·2 million individuals in the USA between 2011 and 2015: a cross-sectional study. Lancet Psychiatry 5, 739–746. 10.1016/S2215-0366(18)30227-X 30099000

[B12] Chiba I. Lee S. Bae S. Makino K. Shinkai Y. Katayama O. . (2022). Isotemporal substitution of sedentary behavior with moderate to vigorous physical activity is associated with lower risk of disability: a prospective longitudinal cohort study. Physical Therapy 102:pzac002. 10.1093/ptj/pzac002 35079837

[B13] Curtis R. G. Dumuid D. McCabe H. Singh B. Ferguson T. Maher C. (2023). The association between 24-hour activity, sedentary and sleep compositions and mental health in Australian adults: a cross-sectional study. J. Activity Sedentary Sleep Behav. 2:15. 10.1186/s44167-023-00024-6 40217512 PMC11960370

[B14] de Faria F. R. Barbosa D. Howe C. A. Canabrava K. L. R. Sasaki J. E. Dos Santos Amorim P. R. (2022). Time-use movement behaviors are associated with scores of depression/anxiety among adolescents: a compositional data analysis. PLoS ONE 17:e0279401. 10.1371/journal.pone.0279401 36584176 PMC9803290

[B15] Dillon C. McMahon E. O'Regan G. Perry I. (2018). Associations between physical behavior patterns and levels of depressive symptoms, anxiety and well-being in middle-aged adults: a cross-sectional study using isotemporal substitution models. BMJ open. 8:e018978. 10.1136/bmjopen-2017-018978 29358436 PMC5781191

[B16] Dishman R. K. McDowell C. P. Herring M. P. (2021). Customary physical activity and odds of depression: a systematic review and meta-analysis of 111 prospective cohort studies. Br. J. Sports Med. 55, 926–934. 10.1136/bjsports-2020-103140 33402345

[B17] Dorothea D. Željko P. Tyman Everleigh S. Josep-Antoni M.-F. Karel H. Carol A. . (2017). The compositional isotemporal substitution model: a method for estimating changes in a health outcome for reallocation of time between sleep, physical activity and sedentary behaviour. Stat. Methods Med. Res. 28, 846–857. 10.1177/0962280217737805 29157152

[B18] Egger M. Smith G. D. Schneider M. Minder C. (1997). Bias in meta-analysis detected by a simple, graphical test. BMJ 315, 629–634. 10.1136/bmj.315.7109.629 9310563 PMC2127453

[B19] Fairclough S. J. Noonan R. J. (2020). “Introduction to physical activity measurement, in *The Routledge Handbook of Youth Physical Activity*. New York, NY: Routledge, 251–260. 10.4324/9781003026426-16

[B20] Filiou M. Sandi C. (2019). Anxiety and brain mitochondria: a bidirectional crosstalk. Trends Neurosci. 42, 573–588. 10.1016/j.tins.2019.07.002 31362874

[B21] Gilchrist J. D. Battista K. Patte K. A. Faulkner G. Carson V. Leatherdale S. T. (2021). Effects of reallocating physical activity, sedentary behaviors, and sleep on mental health in adolescents. Ment. Health Phys. Act. 20:100380. 10.1016/j.mhpa.2020.100380

[B22] Goldfarb A. Jamurtas A. Kamimori G. Hegde S. Otterstetter R. Brown D. (1998). Gender effect on beta-endorphin response to exercise. Med. Sci. Sports Exerc. 30, 1672–1676. 10.1097/00005768-199812000-00003 9861598

[B23] Grgic J. Dumuid D. Bengoechea E. G. Shrestha N. Bauman A. Olds T. . (2018). Health outcomes associated with reallocations of time between sleep, sedentary behavior, and physical activity: a systematic scoping review of isotemporal substitution studies. Int. J. Behav. Nutr. Phys. Act. 15:69. 10.1186/s12966-018-0691-3 30001713 PMC6043964

[B24] Guo Z. Li R. Lu S. (2022). Leisure-time physical activity and risk of depression: a dose-response meta-analysis of prospective cohort studies. Medicine 101:e29917. 10.1097/MD.0000000000029917 35905243 PMC9333473

[B25] Hallgren M. Nguyen T. T. Owen N. Stubbs B. Vancampfort D. Lundin A. . (2020). Cross-sectional and prospective relationships of passive and mentally active sedentary behaviors and physical activity with depression. Br. J. Psychiatry. 217, 413–419. 10.1192/bjp.2019.60 30895922

[B26] Hardy R. J. Thompson S. G. (1996). A likelihood approach to meta-analysis with random effects. *Stat*. Med. 15, 619–629. 10.1002/(SICI)1097-0258(19960330)15:6<619::AID-SIM188>3.0.CO;2-A 8731004

[B27] Higgins J. P. Thomas J. Chandler J. Cumpston M. Li T. Page M. J. . (2019). Cochrane Handbook for Systematic Reviews of Interventions. John Weiley and Sons: Chichester, UK. 10.1002/9781119536604 PMC10284251 31643080

[B28] Higgins J. P. Thompson S. G. (2002). Quantifying heterogeneity in a meta-analysis. Stat. Med. 21, 1539–1558. 10.1002/sim.1186 12111919

[B29] Hofman A. Voortman T. Ikram M. A. Luik A. I. (2022). Substitutions of physical activity, sedentary behaviour and sleep: associations with mental health in middle-aged and elderly persons. J. Epidemiol. Community Health. 76, 175–181. 10.1136/jech-2020-215883 34301796 PMC8762024

[B30] Kandola A. Del Pozo Cruz B. Hayes J. F. Owen N. Dunstan D. W. Hallgren M. (2022). Impact on adolescent mental health of replacing screen-use with exercise: a prospective cohort study. BMJ Open. 301, 240–247. 10.1016/j.jad.2021.12.064 34999126

[B31] Kessler R. C. Bromet E. J. (2013). The epidemiology of depression across cultures. Annu. Rev. Public Health. 34, 119–138. 10.1146/annurev-publhealth-031912-114409 23514317 PMC4100461

[B32] Knapen J. Vancampfort D. Moriën Y. Marchal Y. (2015). Exercise therapy improves both mental and physical health in patients with major depression. Disabil. Rehabil. 37, 1490–1495. 10.3109/09638288.2014.972579 25342564

[B33] Kokkinos P. (2012). Physical activity, health benefits, and mortality risk. ISRN Cardiol. 2012:718789. 10.5402/2012/718789 23198160 PMC3501820

[B34] Larun L. Nordheim L. V. Ekeland E. Hagen K. B. Heian F. (2006). Exercise in prevention and treatment of anxiety and depression among children and young people. Cochrane Database Syst. Rev. 10.1002/14651858.CD004691.pub2 16856055 PMC12742371

[B35] Liu Y. Lin H. Zhang H. Zhang X. Yin S. (2023). Correlation analysis between physical activity and depressive tendencies among occupational groups: an isotemporal substitution approach. BMC Public Health. 23:2241. 10.1186/s12889-023-17134-0 37964346 PMC10644550

[B36] Mammen G. Faulkner G. (2013). Physical activity and the prevention of depression: a systematic review of prospective studies. Am. J. Prev. Med. 45, 649–657. 10.1016/j.amepre.2013.08.001 24139780

[B37] Mathers C. D. Loncar D. (2006). Projections of global mortality and burden of disease from 2002 to 2030. PLoS Med. 3:e442. 10.1371/journal.pmed.0030442 17132052 PMC1664601

[B38] Mekary R. A. Lucas M. Pan A. Okereke O. I. Willett W. C. Hu F. B. . (2013). Isotemporal substitution analysis for physical activity, television watching, and risk of depression. Am. J. Epidemiol. 178, 474–483. 10.1093/aje/kws590 23785112 PMC3727339

[B39] Mekary R. A. Willett W. C. Hu F. B. Ding E. L. (2009). Isotemporal substitution paradigm for physical activity epidemiology and weight change. Am. J. Epidemiol. 170, 519–527. 10.1093/aje/kwp163 19584129 PMC2733862

[B40] Meneguci J. Galvao L. L. Tribess S. Meneguci C. A. G. Virtuoso Junior J. S. (2024). Isotemporal substitution analysis of time between sleep, sedentary behavior, and physical activity on depressive symptoms in older adults: a cross-sectional study. Sao Paulo Med. J. 142:e2023144. 10.1590/1516-3180.2023.0144.r2.04122023 38511771 PMC10950321

[B41] Molendijk M. Molero P. Ortuño Sánchez-Pedreño F. Van der Does W. Angel Martínez-González M. (2018). Diet quality and depression risk: a systematic review and dose-response meta-analysis of prospective studies. J. Affect. Disord. 226, 346–354. 10.1016/j.jad.2017.09.022 29031185

[B42] Monteagudo P. Beltran-Valls M. R. Adelantado-Renau M. Moliner-Urdiales D. (2023). Observational longitudinal association between waking movement behaviors and psychological distress among adolescents using isotemporal analysis: DADOS study. J. Sports Sci. 41, 1290–1298. 10.1080/02640414.2023.2268359 37851923

[B43] Nam H. K. Park J. Cho S. I. (2023). Association between depression, anemia and physical activity using isotemporal substitution analysis. BMC Public Health 23:2236. 10.1186/s12889-023-17117-1 37957654 PMC10644608

[B44] Page M. J. McKenzie J. E. Bossuyt P. M. Boutron I. Hoffmann T. C. Mulrow C. D. . (2021). Updating guidance for reporting systematic reviews: development of the PRISMA 2020 statement. J. Clin. Epidemiol. 134, 103–112. 10.1016/j.jclinepi.2021.02.003 33577987

[B45] Park J. Nam H. K. Cho S. I. (2024). Association between accelerometer-derived physical activity and depression: a cross-sectional study using isotemporal substitution analysis. BMJ Open 14:e078199. 10.1136/bmjopen-2023-078199 38684272 PMC11057242

[B46] Pearce M. Garcia L. Abbas A. Strain T. Schuch F. B. Golubic R. . (2022). Association between physical activity and risk of depression: a systematic review and meta-analysis. JAMA Psychiatry 79, 550–559. 10.1001/jamapsychiatry.2022.0609 35416941 PMC9008579

[B47] Piercy K. L. Troiano R. P. (2018). Physical activity guidelines for Americans from the US department of health and human services: cardiovascular benefits and recommendations. Circ. Cardiovasc. Qual. Outcomes. 11:e005263. 10.1161/CIRCOUTCOMES.118.005263 30571339

[B48] Poudevigne M. S. O'Connor P. J. (2006). A review of physical activity patterns in pregnant women and their relationship to psychological health. Sports Med. 36, 19–38. 10.2165/00007256-200636010-00003 16445309

[B49] Rethorst C. D. Moncrieft A. E. Gellman M. D. Arredondo E. M. Buelna C. Castañeda S. F. . (2017). Isotemporal analysis of the association of objectively measured physical activity with depressive symptoms: results from hispanic community health study/study of latinos (HCHS/SOL). J. Phys. Act. Health. 14, 733–739. 10.1123/jpah.2016-0648 28422609 PMC5794338

[B50] Rong F. Li X. Jia L. Liu J. Li S. Zhang Z. . (2024). Substitutions of physical activity and sedentary behavior with negative emotions and sex difference among college students. Psychol. Sport Exerc. 72:102605. 10.1016/j.psychsport.2024.102605 38346583

[B51] Rosenbaum S. Tiedemann A. Sherrington C. Curtis J. Ward P. B. (2014). Physical activity interventions for people with mental illness: a systematic review and meta-analysis. J. Clin. Psychiatry 75:14465. 10.4088/JCP.13r08765 24813261

[B52] Sadarangani K. P. Schuch F. B. De Roia G. Martinez-Gomez D. Chavez R. Lobo P. . (2023). Exchanging screen for non-screen sitting time or physical activity might attenuate depression and anxiety: a cross-sectional isotemporal analysis during early pandemics in South America. J. Sci. Med. Sport 26, 309–315. 10.1016/j.jsams.2023.04.007 37210319 PMC10118065

[B53] Sampasa-Kanyinga H. Colman I. Dumuid D. Janssen I. Goldfield G. S. Wang J. L. . (2021). Longitudinal association between movement behaviors and depressive symptoms among adolescents using compositional data analysis. PLoS ONE 16:e0256867. 10.1371/journal.pone.0256867 34469485 PMC8409652

[B54] Schuch F. B. Vancampfort D. Firth J. Rosenbaum S. Ward P. B. Silva E. S. . (2018). Physical activity and incident depression: a meta-analysis of prospective cohort studies. Am. J. Psychiatry 175, 631–648. 10.1176/appi.ajp.2018.17111194 29690792

[B55] Shrestha N. Grgic J. Wiesner G. Parker A. Podnar H. Bennie J. . (2019). Effectiveness of interventions for reducing non-occupational sedentary behavior in adults and older adults: a systematic review and meta-analysis. Br. J. Sports Med. 53, 1206–1213. 10.1136/bjsports-2017-098270 29331992

[B56] Singh B. Olds T. Curtis R. Dumuid D. Virgara R. Watson A. . (2023). Effectiveness of physical activity interventions for improving depression, anxiety and distress: an overview of systematic reviews. Br. J. Sports Med. 57, 1203–1209. 10.1136/bjsports-2022-106195 36796860 PMC10579187

[B57] Tang B.-q. Chen B-h. Li Y.-y. Liu H.-q. Xu S.-q. Wang S.-m. (2024). A cross-sectional study on the relationship between 24-hour activities and depressive symptoms in vocational school students. Fudan Univ. J. Med. Sci. 51, 159–165.

[B58] Toseeb U. Brage S. Corder K. Dunn V. J. Jones P. B. Owens M. . (2014). Exercise and depressive symptoms in adolescents: a longitudinal cohort study. JAMA pediatr. 168, 1093–1100. 10.1001/jamapediatrics.2014.1794 25317674

[B59] Tremblay M. S. Carson V. Chaput J.-P. Connor Gorber S. Dinh T. Duggan M. . (2016). Canadian 24-hour movement guidelines for children and youth: an integration of physical activity, sedentary behavior, and sleep. Appl. Physiol. Nutr. Metab. 41, S311–S327. 10.1139/apnm-2016-0203 27306437

[B60] Tremblay M. S. Colley R. C. Saunders T. J. Healy G. N. Owen N. (2010). Physiological and health implications of a sedentary lifestyle. Appl. Physiol. Nutr. Metab. 35, 725–740. 10.1139/H10-079 21164543

[B61] Tully M. A. McMullan I. Blackburn N. E. Wilson J. J. Bunting B. Smith L. . (2020). Sedentary behavior, physical activity, and mental health in older adults: An isotemporal substitution model. Scand. J. Med. Sci. Sports. 30, 1957–1965. 10.1111/sms.13762 32643826

[B62] Vos T. Allen C. Arora M. Barber R. M. Bhutta Z. A. Brown A. . (2016). Global, regional, and national incidence, prevalence, and years lived with disability for 310 diseases and injuries, 1990–2015: a systematic analysis for the global burden of disease study 2015. Lancet 388, 1545–1602. 10.1016/S0140-6736(16)31678-6 27733282 PMC5055577

[B63] Weggemans R. M. Backx F. J. Borghouts L. Chinapaw M. Hopman M. T. Koster A. . (2018). The 2017 Dutch physical activity guidelines. Int. J. Behav. Nutr. Phys. Act. 15, 1–12. 10.1186/s12966-018-0661-9 29940977 PMC6016137

[B64] Wei J. Xie L. Song S. Wang T. Li C. (2019). Isotemporal substitution modeling on sedentary behaviors and physical activity with depressive symptoms among older adults in the U.S.: the national health and nutrition examination survey, 2007–2016. J. Affect Disord. 257, 257–262. 10.1016/j.jad.2019.07.036 31301629

[B65] Wells G. A. Shea B. O'Connell D. Peterson J. Welch V. Losos M. . (2000). The Newcastle-Ottawa Scale (NOS) for Assessing the Quality of Nonrandomized Studies in Meta-analysis. Available online at: https://www.ohri.ca/programs/clinical_epidemiology/oxford.asp (accessed August 15, 2024).

[B66] Wheeler M. Green D. Ellis K. Cerin E. Heinonen I. Naylor L. . (2020). Distinct effects of acute exercise and breaks in sitting on working memory and executive function in older adults: a three-arm, randomised cross-over trial to evaluate the effects of exercise with and without breaks in sitting on cognition. Br. J. Sports Med. 54, 776–781. 10.1136/bjsports-2018-100168 31036563

[B67] World Health Organization. (2017). Depression and Other Common Mental Disorders: Global Health Estimates. Available online at: https://www.who.int/publications/i/item/depression-global-health-estimates (accessed August 15, 2024).

[B68] World Health Organization. (2020). Global Recommendations on Physical Activity for Health. Available online at: https://www.who.int/publications/i/item/9789241599979 (accessed August 15, 2024).

[B69] Yasunaga A. Shibata A. Ishii K. Koohsari M. J. Oka K. (2018). Cross-sectional associations of sedentary behavior and physical activity on depression in Japanese older adults: an isotemporal substitution approach. BMJ Open. 8:e022282. 10.1136/bmjopen-2018-022282 30257848 PMC6169747

[B70] Zhou Q. Guo C. Yang X. He N. (2023). Dose-response association of total sedentary behavior and television watching with risk of depression in adults: a systematic review and meta-analysis. J Affect Disord. 324, 652–659. 10.1016/j.jad.2022.12.098 36610602

[B71] Zhou Y. Huang Z. Liu Y. Liu D. (2024). The effect of replacing sedentary behavior with different intensities of physical activity on depression and anxiety in Chinese university students: an isotemporal substitution model. BMC Public Health 24:1388. 10.1186/s12889-024-18914-y 38783202 PMC11118727

[B72] Zhu J. H. Shen Z. Z. Liu B. P. Jia C. X. (2024). Replacement of sedentary behavior with various physical activities and the risk of incident depression: a prospective analysis of accelerator-measured and self-reported UK Biobank data. Soc. Psychiatry Psychiatr. Epidemiol. 59, 2105–2116. 10.1007/s00127-024-02708-z 39001888

