# Supplementary material for: Association of reallocating time between physical activity and sedentary behavior on the risk of depression: a systematic review and meta-analysis
Source: Front Psychol. 2025 Apr 30;16:1505061. doi: 10.3389/fpsyg.2025.1505061 (PMC12075196; doi:10.3389/fpsyg.2025.1505061)
Supplement: Supplementary file 1 [file Table_1.docx]

# Supplementary Material Table S1-8.

## **Table S.1 Search strategy in PubMed (2024 June 15th)**

| **#** | Query | Results |
| --- | --- | --- |
| **#4** | #1 AND #2 AND #3 | \| **121** \| \| --- \| |
| **#3** | ((((((((Reallocation [MeSH Terms]) OR (Replace)) OR (Time-use)) OR (Substitutions)) OR (Isotemporal substitution)) OR (Joint)) OR (24 Time-use)) OR (Replacement)) OR (Substitute) | **1,424** |
| **#2** | "Exercise"[MeSH Terms] OR ("Exercises"[Title/Abstract] OR "physical activity"[Title/Abstract] OR "activities physical"[Title/Abstract] OR "activity physical"[Title/Abstract] OR "physical activities"[Title/Abstract] OR "exercise physical"[Title/Abstract] OR "exercises physical"[Title/Abstract] OR "physical exercise"[Title/Abstract] OR "physical exercises"[Title/Abstract] OR "acute exercise"[Title/Abstract] OR "acute exercises"[Title/Abstract] OR "exercise acute"[Title/Abstract] OR "exercises acute"[Title/Abstract] OR "exercise isometric"[Title/Abstract] OR "exercises isometric"[Title/Abstract] OR "isometric exercises"[Title/Abstract] OR "isometric exercise"[Title/Abstract] OR "exercise aerobic"[Title/Abstract] OR "aerobic exercise"[Title/Abstract] OR "aerobic exercises"[Title/Abstract] OR "exercises aerobic"[Title/Abstract] OR "exercise training"[Title/Abstract] OR "exercise trainings"[Title/Abstract] OR "training exercise"[Title/Abstract] OR (("education"[MeSH Subheading] OR "education"[All Fields] OR "Training"[All Fields] OR "education"[MeSH Terms] OR "train"[All Fields] OR "train s"[All Fields] OR "trained"[All Fields] OR "training s"[All Fields] OR "Trainings"[All Fields] OR "trains"[All Fields]) AND "Exercise"[Title/Abstract])) OR ("sedentary behavior"[MeSH Terms] OR ("behavior sedentary"[Title/Abstract] OR "sedentary behaviors"[Title/Abstract] OR "sedentary lifestyle"[Title/Abstract] OR "lifestyle sedentary"[Title/Abstract] OR "physical inactivity"[Title/Abstract] OR "inactivity physical"[Title/Abstract] OR "lack of physical activity"[Title/Abstract] OR "sedentary time"[Title/Abstract] OR "sedentary times"[Title/Abstract] OR "time sedentary"[Title/Abstract])) | **458,463** |
| **#1** | "depressive disorder"[MeSH Terms] OR "Depression"[MeSH Terms] OR "depressive symptoms"[Title/Abstract] OR "depressive symptom"[Title/Abstract] OR "symptom depressive"[Title/Abstract] OR "emotional depression"[Title/Abstract] OR "depression emotional"[Title/Abstract] | **291,745** |
| **Table S.2 Search strategy in Scopus (2024 June 15th)** | |  |
| # | Query | Results |
| **S4** | S1 AND S2 AND S3 | 183 |
| **S3** | All field: " Joint or Isotemporal substitution or Substitutions or Time-use or Replace or Reallocation " | **161,5** |
| **S2** | Article title, Abstact, keywords: "Physical Activity" or "Activities, Physical" or "Activity, Physical" or "Physical Activities" or "Exercise, Physical" or "Exercises, Physical" or "Physical Exercise" or "Physical Exercises" or "Acute Exercise" or "Acute Exercises" or "Exercise, Acute" or "Exercises, Acute" or "Exercise, Isometric" or "Exercises, Isometric" or "Isometric Exercises" or "Isometric Exercise" or "Exercise, Aerobic" or "Aerobic Exercise" or "Aerobic Exercises" or "Exercises, Aerobic" or "Exercise Training" or "Exercise Trainings" or "Training, Exercise" or "Trainings, Exercise" or "Sedentary Behavior" or "Behavior, Sedentary" or "Sedentary Behaviors" or "Sedentary Lifestyle" or "Lifestyle, Sedentary" or "Physical Inactivity" or "Inactivity, Physical" or "Lack of Physical Activity" or "Sedentary Time" or "Sedentary Times" or "Time, Sedentary" | **402,155** |
| **S1** | Article title, Abstact, keywords: "Depression" or "Depressive Symptoms" or "Depressive Symptom" or "Symptom, Depressive" or "Emotional Depression" or "Depression, Emotional" | **944,273** |
| **Table S.3 Search strategy in PsycINFO via EBSCO (2024 June 15th)** | |  |
| # Query | | Results |

| **S4** | S1 AND S2 AND S3 | 23 |
| --- | --- | --- |
| **S3** | TX All Text (Joint or Isotemporal substitution or Substitutions or Time-use or Replace or Reallocation) | **76,89** |
| **S2** | TI (Physical Activity* or "Activities, Physical" or "Activity, Physical" or "Physical Activities*" or "Exercise, Physical" or "Exercises, Physical" or "Physical Exercise" or "Physical Exercises" or "Acute Exercise" or "Acute Exercises" or "Exercise, Acute" or "Exercises, Acute" or "Exercise, Isometric" or "Exercises, Isometric" or "Isometric Exercises" or "Isometric Exercise" or "Exercise, Aerobic" or "Aerobic Exercise" or "Aerobic Exercises" or "Exercises, Aerobic" or "Exercise Training" or "Exercise Trainings" or "Training, Exercise" or "Trainings, Exercise" or "Sedentary Behavior" or "Behavior, Sedentary" or Sedentary Behaviors* or "Sedentary Lifestyle" or "Lifestyle, Sedentary" or "Physical Inactivity" or "Inactivity, Physical" or "Lack of Physical Activity" or "Sedentary Time" or "Sedentary Times" or "Time, Sedentary"  ) OR AB (Physical Activity* or "Activities, Physical" or "Activity, Physical" or "Physical Activities*" or "Exercise, Physical" or "Exercises, Physical" or "Physical Exercise" or "Physical Exercises" or "Acute Exercise" or "Acute Exercises" or "Exercise, Acute" or "Exercises, Acute" or "Exercise, Isometric" or "Exercises, Isometric" or "Isometric Exercises" or "Isometric Exercise" or "Exercise, Aerobic" or "Aerobic Exercise" or "Aerobic Exercises" or "Exercises, Aerobic" or "Exercise Training" or "Exercise Trainings" or "Training, Exercise" or "Trainings, Exercise" or "Sedentary Behavior" or "Behavior, Sedentary" or Sedentary Behaviors* or "Sedentary Lifestyle" or "Lifestyle, Sedentary" or "Physical Inactivity" or "Inactivity, Physical" or "Lack of Physical Activity" or "Sedentary Time" or "Sedentary Times" or "Time, Sedentary"  ) | **154,841** |
| **S1** | TI (Depression* or "Depressive Symptoms" or "Depressive Symptom" or "Symptom, Depressive" or "Emotional Depression" or "Depression, Emotional" ) OR AB (Depression* or "Depressive Symptoms" or "Depressive Symptom" or "Symptom, Depressive" or "Emotional Depression" or "Depression, Emotional" ) | **453,366** |

## **Table S.4 Search strategy in SPORTDiscus via EBSCO (2024 June 15th)**

| # Query | Results |
| --- | --- |

| **S4** | S1 AND S2 AND S3 | 55 |
| --- | --- | --- |
| **S3** | TX All Text (Joint or Isotemporal substitution or Substitutions or Time-use or Replace or Reallocation) | **76,89** |
| **S2** | TI (Physical Activity* or "Activities, Physical" or "Activity, Physical" or "Physical Activities*" or "Exercise, Physical" or "Exercises, Physical" or "Physical Exercise" or "Physical Exercises" or "Acute Exercise" or "Acute Exercises" or "Exercise, Acute" or "Exercises, Acute" or "Exercise, Isometric" or "Exercises, Isometric" or "Isometric Exercises" or "Isometric Exercise" or "Exercise, Aerobic" or "Aerobic Exercise" or "Aerobic Exercises" or "Exercises, Aerobic" or "Exercise Training" or "Exercise Trainings" or "Training, Exercise" or "Trainings, Exercise" or "Sedentary Behavior" or "Behavior, Sedentary" or Sedentary Behaviors* or "Sedentary Lifestyle" or "Lifestyle, Sedentary" or "Physical Inactivity" or "Inactivity, Physical" or "Lack of Physical Activity" or "Sedentary Time" or "Sedentary Times" or "Time, Sedentary"  ) OR AB (Physical Activity* or "Activities, Physical" or "Activity, Physical" or "Physical Activities*" or "Exercise, Physical" or "Exercises, Physical" or "Physical Exercise" or "Physical Exercises" or "Acute Exercise" or "Acute Exercises" or "Exercise, Acute" or "Exercises, Acute" or "Exercise, Isometric" or "Exercises, Isometric" or "Isometric Exercises" or "Isometric Exercise" or "Exercise, Aerobic" or "Aerobic Exercise" or "Aerobic Exercises" or "Exercises, Aerobic" or "Exercise Training" or "Exercise Trainings" or "Training, Exercise" or "Trainings, Exercise" or "Sedentary Behavior" or "Behavior, Sedentary" or Sedentary Behaviors* or "Sedentary Lifestyle" or "Lifestyle, Sedentary" or "Physical Inactivity" or "Inactivity, Physical" or "Lack of Physical Activity" or "Sedentary Time" or "Sedentary Times" or "Time, Sedentary"  ) | **203,348** |
| **S1** | TI (Depression* or "Depressive Symptoms" or "Depressive Symptom" or "Symptom, Depressive" or "Emotional Depression" or "Depression, Emotional"  ) OR AB (Depression* or "Depressive Symptoms" or "Depressive Symptom" or "Symptom, Depressive" or "Emotional Depression" or "Depression, Emotional"  ) | **331,991** |

## **Table S.5 Characteristics of included cohorts**

| **Author(year)** | **Country** | | **Cohort** | **Age(years**） | **No of participants (women**） | **PA types** | **PA measurement** | **FU(years)** | | |
| --- | --- | --- | --- | --- | --- | --- | --- | --- | --- | --- |
| **cohort studies** | | | | | | | | | |  |
| Chiba et al., 2021 | JP | | 3691 participants from LTCI in Japan | 74.0 (5.0) | 3691(57.2%) | SB;LPA;MVPA | Accelerometer | | 2 years |  |
| Monteagudo et al., 2023 | | ESP | 197 participants from 3-year observational research in Spain | 13.9(0.3) | 197(46.2%) | SB;LPA;MVPA | Accelerometer | 3 years | |  |
| Zhu et al., 2024 | CHN | | 103614 participants form UK Biobank aged 37 to 73 years | 56.2(7.8) | 84168(55.4%) | SB;LPA;MVPA | Accelerometer | 3 years | |  |
| Rong et al., 2024 | CHN | | 8723 freshman aged 16–22 years from three colleges in China. | 18.4(0.9) | 8723(39.6%) | SB;LPA;MVPA;VPA | self-report | 0.5 years | |  |
| Hofman et al., 2022 | NL | | 14926 participants from the population-based Rotterdam Study | 70.91(9.26) | 1943(51.6%) | SB;LPA;MVPA | Accelerometer | 5 years | |  |
| Cao et al., 2022 | CHN | | A total of 360,047 participants (aged 37-73 years) in the UK Biobank | 55.8(8.1) | 360047(54.5%) | LPA;MPA;VPA | self-report | 14 years | |  |
| Cabanas-Sánchez et al., 2021 | ESP | | 2489 participants from Madrid and four surrounding cities in Spain | 71.68 | 2489(53.07%) | SB;LPA;MVPA | Accelerometer | 2.31 years | |  |
| Hallgren et al, 2020 | SE | | 43 863 Swedish adults were initially surveyed | 49.2(15.8) | 43863(66%) | SB;LPA;MVPA | self-report | 13 years | |  |
| Mekary et al., 2013 | US | | 32,900 female registered nurses in the United States | 62 | 32900(100%) | SB;LPA;MPA;VPA | self-report | 10 years | |  |
| Sampasa-Kanyinga et al., 2021 | CAN | | 14,620 students from the Canadian provinces of Ontario, British Columbia, Alberta and Quebec | 14.9 | 14620(46%) | MVPA | self-report | 1 years | |  |
| Kandola et al., 2021 | BR | | 60,235 UK Biobank participants | 55.9 (7.7) | 60235(56.0%) | SB;LPA;MVPA | Accelerometer | 2 years | |  |
| **cross-sectional studies** | | | | | | | | | |  |
| Araki et al., 2022 | JP | | 139 frail elderly people who make use of sports-specific day services | 83.1(5.9) | 139(64%) | SB;LPA;MVPA | accelerometer | － | |  |
| Curtis et al., 2023 | AU | | 322 participants recruited in three waves from greater metropolitan Ade- laide, South Australia | 40.4 | 322(58.1%) | SB;LPA;MVPA | Accelerometer | | － |  |
| Park et al., 2024 | KR | | 1543 adults from the Republic of Korea | n/r | 1543(61.6%) | SB;LPA;MPA;VPA | Accelerometer | | － |  |
| Nam et al., 2023 | KR | | 18,622 participants from the Korean National Health and Nutrition Examination Survey | n/r | 18622(n/r) | SB;LPA;MPA;VPA | self-report | | － |  |
| Dillon et al., 2018 | IE | | In total 3807 potential participants were selected from the practice list | 59.6(5.5) | 397(53.9%) | SB;LPA;MVPA | Accelerometer | | － |  |
| Liu et al., 2023 | CHN | | A total of 10656 employees from 79 companies in four provinces of China | 33.1 | 10656(55.6%) | SB;LPA;MPA;VPA | self-report | | － |  |
| Yasunaga et al.，2018 | JP | | A total of 276 older adults aged 65–85 years living in Japan | 77.4 | 276(38%) | SB;LPA;MVPA | Accelerometer | | － |  |
| Gilchrist et al., 2021 | CAN | | a sample of 46413 adolescents participating in the COMPASS Study | n/r | 46413(51.5%) | SB;MVPA | self-report | | － |  |
| Sadarangani et al., 2023 | CL | | The study involved 1,981 adult participants from Chile, Argentina and Brazil | n/r | 1981(76%) | SB;MPA;VPA | self-report | | － |  |
| Rethorst et al., 2017 | US | | The study involved Hispanic/Latino men and women ages 18 to 74, with 11116 participants from four metropolitan areas in the United States | 41.06 | 11116(52.13%) | SB;LPA;MPA;VPA | Accelerometer | | － |  |
| Meneguci et al., 2024 | BR | | the analysis included 473 participants registered in the Family Health Strategy | 70.2 | 473(62.6%) | SB;MVPA | self-report | | － |  |
| Wei et al., 2019 | US | | 8374 participants involved NHANES 2007-2016 | n/r | 8374(50.8%) | SB;LPA;MVPA | self-report | | － |  |
| Tully et al.,2020 | UK | | 2660 older adults from the SITLESS study, which is a prospective trial of community-dwelling men and women aged ≥65 years from Denmark, Spain, Germany, and Northern Ireland | 75.18 (6.17) | 1360(61.8%) | SB;LPA;MVPA | accelerometer | | － |  |
| Zhou et al., 2024 | CHN | | 446 university students from Hubei Province,Zhejiang Province, and Shanghai | 21.13（3.53） | 318(60.1%） | SB;LPA;MVPA | accelerometer | | － |  |
| de Faria et al., 2022 | BR | | the technical high school of the Federal Institute of Education, Science, and Technology of Triaˆngulo Mineiro, Ituiutaba Campus, Minas Gerais, Brazil. | 16.08（0.95） | 217(49.3%） | SB;LPA;MVPA | accelerometer | | － |  |
| Tang et al., 2024 | CHN | | 10086 participants from grade one to grade three students attending vocational schools in Shanghai and Jiangsu Province | 16.70（1.19） | 8149(43.01%) | SB;LPA;MPA;VPA | self-report | | － |  |
| FU: follow up.SD: standard deviation. n/a: not applicable. n/r: not reported.SB: sedentary behavior. LPA: light physical activity. MVPA: moderate-to-vigorous physical activity. TPA: total physical activity. LIPA: light-intensity physical activity. LMVPA: light plus moderate-to-vigorous activity. METs: Metabolic Equivalents of Task. cpm: counts per minute. h/d: hours per day. min/day: minutes per day. mG: milli-G.Q=quartile. T=tertile. S=subgroup. IQR: Interquartile Range. Mdn: median M: in male. F: in female.CA:Canada.SE:Sweden.UK:UnitedKingdom.AU:Australia.BR:Brazil.DE:Germany.TW:Taiwan.NL:Netherlands.PL:Poland.JP:Japan.CN:China.KR:Korea.ES:Spain.USA:United States of America.GR:Greece.uni.:university.LABS-2:Longitudinal Assessment of Bariatric Surgery-2.Birth Cohort 1936:Participants were drawn from the Lothian Birth Cohort 1936(LBC1936).Birth Cohort 1950:The West of Scotland Twenty-07 1950s.NHANES:7162 participants from the 2005-2006 National Health and Nutrition Examination Survey. NHANES: National Health and Nutrition Examination Survey 2005–2006.Project MOBILE: Measuring Our Behaviors in Living Environments. SHARE: the Survey of Health, Ageing and Retirement in Europe. HCHS/SOL:16,415 Hispanic/Latino adults. NHANES: National Health and Nutrition Examination Survey. JNAO: Japan's National Astronomical Observatory. | | | | | | | | | |  |

**Table S6 Quality assessment of included studies (Newcastle Ottawa Scale)**

|  | | | | | | | | | | | | | | | | | | | | | | |
| --- | --- | --- | --- | --- | --- | --- | --- | --- | --- | --- | --- | --- | --- | --- | --- | --- | --- | --- | --- | --- | --- | --- |
| cross-sectional studies | Selection | | | | | | | | | | | | | Comparability | | Outcome | | | | | | Quality score（10） |
| Author (year) | Representativeness of the sample（*） | | | | Sample size（*） | | | Non-respondents（*） | | | Ascertainment of the exposure (risk factor)（**） | | | Comparability and control of confounding factors（**） | | Assessment of outcome（**） | | | | Statistical test（*） | |  |
|  | a | b | c | d | a | b | c | a | b | c | a | b | c | a | b | a | b | c | d | a | b |  |
| Araki et al.，2022 |  | * |  |  |  | － |  | * |  |  | ** |  |  | ** |  |  |  | * |  | * |  | 9 |
| Curtis et al., 2023 |  | * |  |  | * |  |  | * |  |  | ** |  |  | ** |  |  |  | * |  | * |  | 9 |
| Park et al.，2024 | * |  |  |  | * |  |  | * |  |  | ** |  |  | ** |  |  |  | * |  | * |  | 9 |
| Nam et al., 2023 |  | * |  |  | * |  |  | * |  |  |  | * |  | ** |  |  |  | * |  | * |  | 8 |
| Dillon et al., 2018 |  | * |  |  | * |  |  | * |  |  | ** |  |  | ** |  |  |  | * |  | * |  | 9 |
| Liu et al., 2023 |  | * |  |  | * |  |  | * |  |  |  | * |  | ** |  |  |  | * |  | * |  | 8 |
| Yasunaga et al.，2018 |  | * |  |  | * |  |  | * |  |  | ** |  |  | ** |  |  |  | * |  | * |  | 9 |
| Gilchrist et al., 2021 |  | * |  |  | * |  |  | * |  |  |  | * |  | ** |  |  |  | * |  | * |  | 8 |
| Sadarangani et al., 2023 |  | * |  |  | * |  |  | * |  |  |  | * |  | ** |  |  |  | * |  | * |  | 8 |
| Rethorst et al., 2017 | * |  |  |  | * |  |  | * |  |  | ** |  |  | ** |  |  |  | * |  | * |  | 9 |
| Meneguci et al., 2024 | * |  |  |  | * |  |  | * |  |  |  | * |  | ** |  |  |  | * |  | * |  | 8 |
| Wei et al., 2019 | * |  |  |  | * |  |  | * |  |  |  | * |  | ** |  |  |  | * |  | * |  | 8 |
| Tully et al.,2020 |  | * |  |  | * |  |  | * |  |  | ** |  |  | ** |  |  |  | * |  | * |  | 9 |
| Zhou et al.，2024 |  | * |  |  | * |  |  |  | － |  | * |  |  | ** |  |  |  | * |  | * |  | 7 |
| de Faria et al., 2022 |  | * |  |  | * |  |  |  | － |  | ** |  |  | ** |  |  |  | * |  | * |  | 8 |
| Tang et al., 2024 |  | * |  |  | * |  |  | * |  |  |  | * |  | ** |  |  |  | * |  | * |  | 8 |
| cohort study | Selection | | | | | | | | | | | | | Comparability | | Outcome | | | | | | Quality score（9） |
| Author (year) | Representativeness of the exposed cohort (adult mixed gender or male or female)（*） | | | | Selection of the non-exposed cohort（*） | | | Ascertainment of exposure（*） | | | Demonstration that outcome of interest was not present at start of study（*） | | | Comparability of cohorts on the basis of the design or analysis（**） | | Assessment of outcome（*） | | Was follow-up long enough for outcomes to occur（*） | | Adequacy of follow up of（*） | |  |
| Cao et al., 2022 | * | | | | * | | | * | | | * | | | ** | | * | | * | | － | | 8 |
| Cabanas‑Sánchez et al., 2021 | * | | | | － | | | * | | | * | | | ** | | * | | * | | * | | 8 |
| Hallgren et al, 2020 | * | | | | － | | | － | | | * | | | * | | * | | * | | * | | 6 |
| Mekary et al., 2013 | * | | | | － | | | * | | | * | | | * | | * | | * | | － | | 6 |
| Sampasa-Kanyinga et al., 2021 | * | | | | * | | | － | | | * | | | * | | * | | － | | － | | 5 |
| Monteagudo et al.,2023 | * | | | | * | | | * | | | * | | | ** | | * | | * | | － | | 7 |
| Zhu et al,. 2024 | * | | | | * | | | * | | | * | | | ** | | * | | * | | － | | 8 |
| Rong et al.,2024 | * | | | | * | | | * | | | * | | | ** | | * | | － | | * | | 8 |
| Hofman et al., 2022 | * | | | | * | | | * | | | * | | | ** | | * | | * | | － | | 8 |
| Chiba et al., 2021 | * | | | | * | | | * | | | * | | | ** | | * | | * | | * | | 9 |
| Kandola et al., 2021 | * | | | | * | | | * | | | * | | | ** | | * | | * | | * | | 9 |

# Supplementary Material Fig. 1-4.
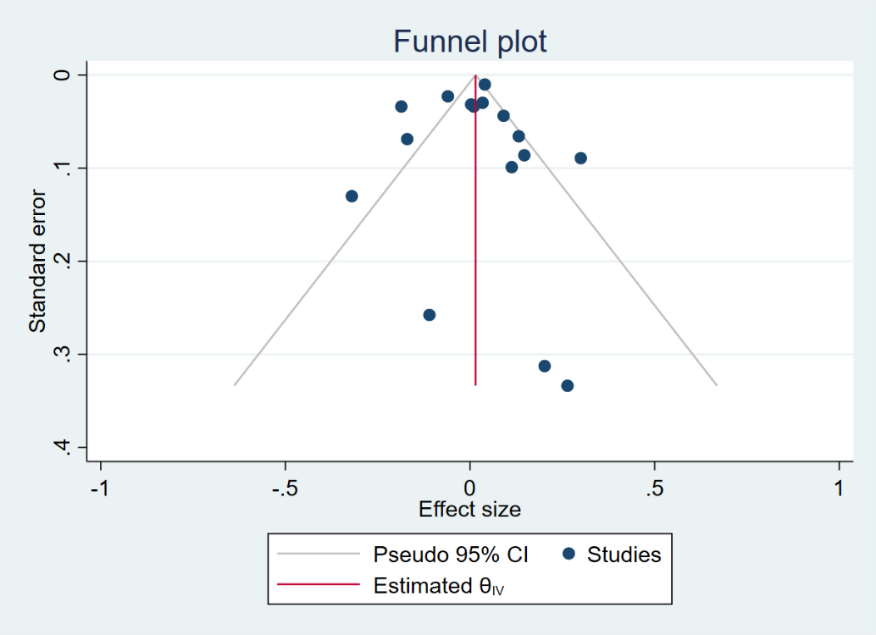


Fig. 1 funnel plot for Reallocating Sedentary Time to LPA


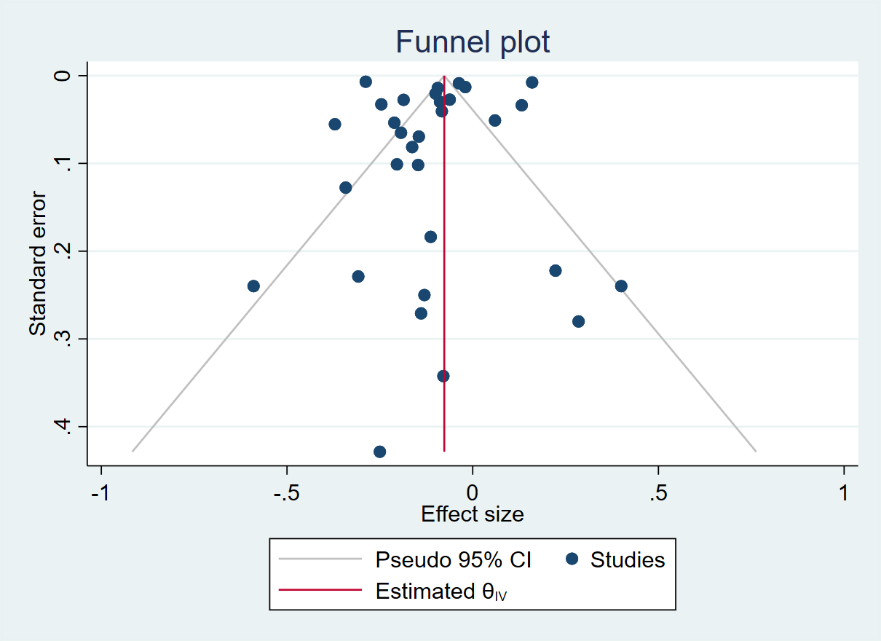


Fig. 2 funnel plot for Reallocating Sedentary Time to MVPA


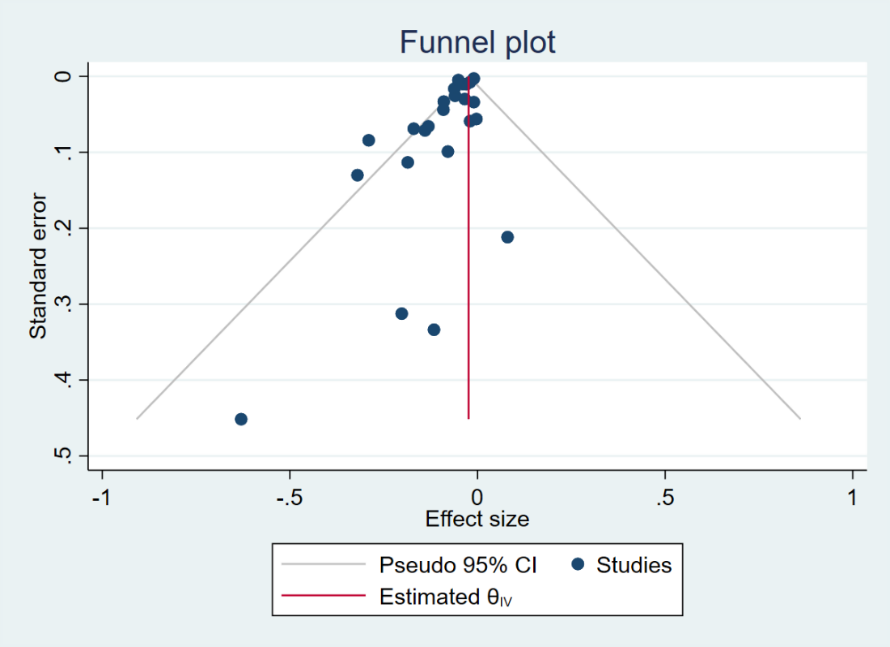


Fig. 3 funnel plot for Reallocating LPA to Sedentary Time


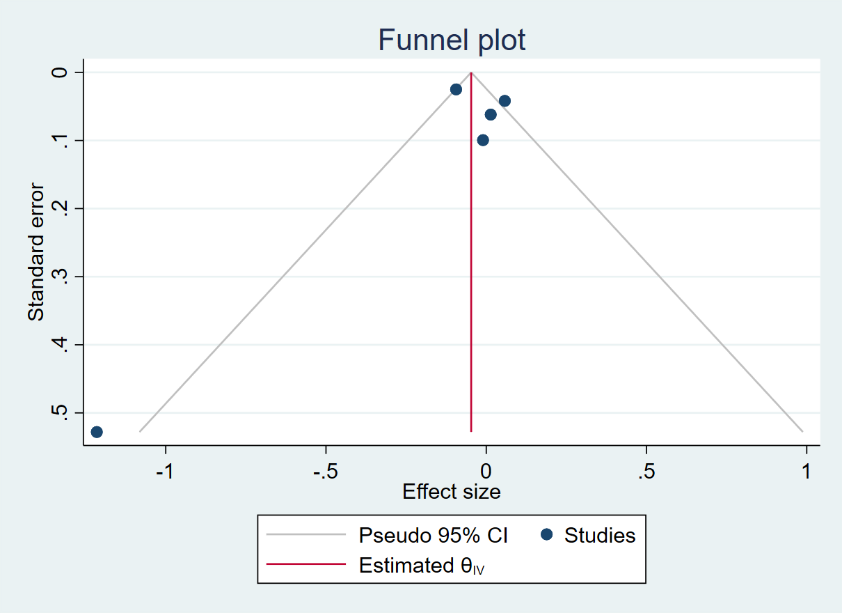


Fig. 4 funnel plot for Reallocating MVPA to Sedentary Time
